# Fast and selective reduction of nitroarenes under visible light with an earth-abundant plasmonic photocatalyst

**DOI:** 10.1038/s41565-022-01087-3

**Published:** 2022-03-28

**Authors:** Aby Cheruvathoor Poulose, Giorgio Zoppellaro, Ioannis Konidakis, Efthymis Serpetzoglou, Emmanuel Stratakis, Ondřej Tomanec, Matthias Beller, Aristides Bakandritsos, Radek Zbořil

**Affiliations:** 1 Regional Centre of Advanced Technologies and Materials, Czech Advanced Technology and Research Institute, Palacký University, Olomouc, Czech Republic; 2 Institute of Electronic Structure and Laser Foundation for Research and Technology-Hellas, Heraklion, Greece; 3 Leibniz-Institute for Catalysis, Rostock, Germany; 4 Nanotechnology Centre, Centre of Energy and Environmental Technologies, VŠB–Technical University of Ostrava, Ostrava-Poruba, Czech Republic

**Keywords:** Photocatalysis, Nanoparticles, Heterogeneous catalysis

## Abstract

Reduction of nitroaromatics to the corresponding amines is a key process in the fine and bulk chemicals industry to produce polymers, pharmaceuticals, agrochemicals and dyes. However, their effective and selective reduction requires high temperatures and pressurized hydrogen and involves noble metal-based catalysts. Here we report on an earth-abundant, plasmonic nano-photocatalyst, with an excellent reaction rate towards the selective hydrogenation of nitroaromatics. With solar light as the only energy input, the chalcopyrite catalyst operates through the combined action of hot holes and photothermal effects. Ultrafast laser transient absorption and light-induced electron paramagnetic resonance spectroscopies have unveiled the energy matching of the hot holes in the valence band of the catalyst with the frontier orbitals of the hydrogen and electron donor, via a transient coordination intermediate. Consequently, the reusable and sustainable copper-iron-sulfide (CuFeS_2_) catalyst delivers previously unattainable turnover frequencies, even in large-scale reactions, while the cost-normalized production rate stands an order of magnitude above the state of the art.

## Abstract (web summary)

A low-cost plasmonic photocatalyst based on earth-abundant metals (Fe, Cu) maximizes solar energy conversion due to the concerted interplay of energies and interactions between reactants and hot carriers, thus producing aromatic amines with a high yield.

## Main

The effective transformation of organics into high added-value compounds is one of the pillars of a technologically advanced society^1,2^. The reduction of nitroaromatics into amines, for example, is considered the key intermediate stage in the synthesis of dyes, polymers and many life-science products including antioxidants, pharmaceuticals and agrochemicals^3^. In general, aniline derivatives are synthesized industrially by the hydrogenation of nitroaromatics using noble metal-based thermal catalysts and H_2_ pressurized gas as a reducing agent^4^, making such processes costly and potentially hazardous. Therefore, identifying sustainable catalysts with high activity for the reduction of nitroarenes under safer and eco-friendly conditions is a great challenge.

Advancements in reduction technologies of nitroarenes have demonstrated promising noble metal-free (photo)catalysts, such as transition metal oxides (Fe_2_O_3_ (ref. ^5^), Co_3_O_4_ (ref. ^6^), Cu_2_O (ref. ^7^)), sulfides (Cu_2_S (ref. ^8^) and CdS (ref. ^9^)), carbon-embedded metal species (Fe^0^/graphene-oxide^10^, Ni (ref. ^11^), Co (refs. ^12,13^)) and coordination complexes (Fe-bipyridine^14^, Zn-based metal organic framework^15^). However, there are still limitations associated with low selectivity and reaction rates, with elevated reaction temperatures and irradiation intensities, need for pressurized H_2_, long reaction times and limited recyclability^5–7,9,11,16,17^ (Supplementary Table 1).

Over the past decade, new insights into plasmon-enhanced nanocatalysis for organic transformations have attracted substantial attention offering improved selectivities, enhanced reaction rates and milder reaction conditions^18,19^. Nevertheless, plasmonic catalysts are mostly based on costly noble metals, such as Au, Ag, Pd and Pt (refs. ^18,20–23^). Furthermore, the desirable coordination of the reactants with the surface of such metallic nanoparticles is not favoured due to the low surface reactivity of the latter, thus requiring the construction of multicomponent nanocatalysts^19,24^. Ideally, a plasmonic photocatalyst should be endowed with intense plasmonic features but also with intrinsic catalytic activity through a strong interaction/coordination with the substrates. The second key limitation is related to the short-lived hot carriers and the difficulty of extracting this energy to perform a catalytic function, which currently attracts profound attention^25^. Identifying pathways to effectively channel the energy from the plasmonic catalyst to the substrates is recognized as a critical aspect in achieving enhanced catalytic efficiencies^25,26^. Recently, ternary chalcogenide nanocrystals (NCs) have stimulated research due to their low toxicity, earth abundance and tuneable band gap^27^. Among them, chalcopyrite (CuFeS_2_) is a naturally occurring mineral having a bulk band gap of 0.5 eV and a tetragonal crystal structure, with tetrahedrally coordinated Cu^1+^ and Fe^3+^ ions with sulfur^28,29^. In the nano-form, CuFeS_2_ NCs^29^ exhibit localized surface plasmon resonance at 2.4 eV, resembling gold^30^. CuFeS_2_ NCs are nonemissive^28^, and the excited surface plasmons dominantly relax through nonradiative damping because of the intermediate energy bands^29^, generating hot electrons or holes and heat^28,30^. Considering this and the high coordination proclivity of Fe-S units (ubiquitous in hydrogenases^31^) for hydrogen atoms and other organics, the CuFeS_2_ NCs could represent an attractive plasmonic catalyst in reductive transformations—an aspect that has yet to be explored.

Here we report that CuFeS_2_ NCs deliver excellent reaction rates towards the selective hydrogenation of nitroaromatics using hydrazine as a proton and electron donor, bypassing the need for noble metals, elevated temperatures, intense irradiation or H_2_ gas. With solar light as the only energy input, the catalyst operates through the combined action of hot hole/electron formation and photothermal conversion. Ultrafast laser transient absorption and light-induced electron paramagnetic resonance spectroscopies unveiled the energy matching of the catalyst’s electron holes with the highest occupied molecular orbital (HOMO) of hydrazine, activating it for the hydrogenation of the nitro-group into the respective amine. As a result, the plasmonic CuFeS_2_ photocatalyst delivers an outstanding turnover frequency (TOF), while the cost-normalized production rate appears to stand an order of magnitude above the state of the art. The potency of the catalyst is further increased because it keeps its activity even against demanding substrates with sensitive side-groups, as well as after recycling under conditions of its maximum production rate or in large-scale reactions.

## Results and discussion

### Characterization of CuFeS_2_ NCs

**Fig. 1:**
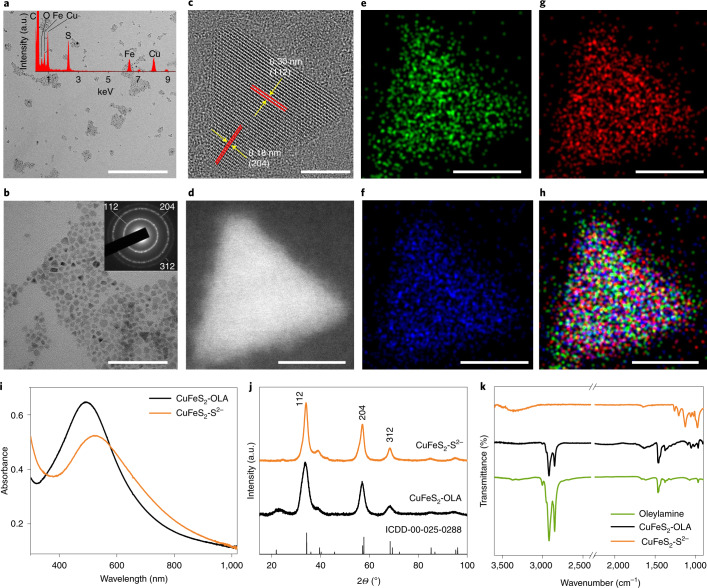
Structural identity of the CuFeS_2_ NCs. **a**,**b**, TEM images of the CuFeS_2_ NCs. Scale bars, 400 nm (**a**); 100 nm (**b**). Insets: the EDS (**a**); the selected area electron diffraction of the NCs (**b**). **c**, High-resolution TEM image of a single NC with marked lattice fringes. Scale bar, 5 nm. **d**–**f**, High-angle annular dark field–scanning TEM image (**d**) of a single NC with the corresponding EDS chemical mapping for Cu (**e**), S (**f**) and Fe (**g**). Scale bars, 8 nm. **h**, Combined mapping for Cu, Fe and S. Scale bar, 8 nm. **i**–**k**, UV–vis absorption spectra (beam path-length, 1﻿ cm) (**i**), XRD analysis (**j**) and FTIR spectra (**k**) of CuFeS_2_ NCs before (CuFeS_2_-OLA) and after (CuFeS_2_-S^2−^) the ligand exchange reaction. OLA, oleylamine. Source data

Oleylamine-capped CuFeS_2_ NCs displayed an average size of 8–10 nm, as indicated by transmission electron microscopy (TEM) (Fig. 1a–c and Supplementary Figs 2 and 3). Energy dispersive X-ray analysis (EDS) (Fig. 1a, inset and Supplementary Fig. 3c) and elemental mapping with high-angle annular dark field–scanning TEM (Fig. 1d–h) confirmed the homogeneous distribution of Cu, Fe and S elements throughout the crystal. The selected area electron diffraction (Fig. 1b, inset) and the X-ray diffraction (XRD) pattern (Fig. 1j) showed the characteristic diffraction rings and reflections, respectively, of the (112), (204) and (312) lattice planes of the tetragonal CuFeS_2_ phase^28^_,_ confirming the purity of the product_._ Ultraviolet–visible light (UV–vis) absorption spectra of the NCs (Fig. 1i) demonstrated broad absorption at 520 nm, attributed to the plasmon resonance of the CuFeS_2_ NCs^28^.

The oleylamine-capping agents of the CuFeS_2_ NCs were exchanged with S^2−^ ions to render them more dispersible in polar solvents and improve the interactions with the reactants. UV-vis absorption spectra before and after the ligand exchange (Fig. 1i) indicated that the plasmonic band was only slightly broadened and red-shifted. XRD (Fig. 1j) and Raman spectra (Supplementary Fig. 4a) also confirmed the preservation of the crystal structure. The successful ligand exchange was confirmed with Fourier transform infrared spectroscopy (FTIR) (Fig. 1k), showing the elimination of the oleylamine spectral features at 2,987 and 2,900 cm^−1^. Similarly, X-ray photoelectron spectroscopy (XPS) (Supplementary Fig. 4b) showed a dramatic reduction—or complete elimination—of the nitrogen peak (circled in red) in the CuFeS_2_-S^2−^ after removal of oleylamine. More details on the XPS characterization are available in Supplementary Fig. 5.

### Photocatalytic performance of CuFeS_2_ NCs

**Fig. 2:**
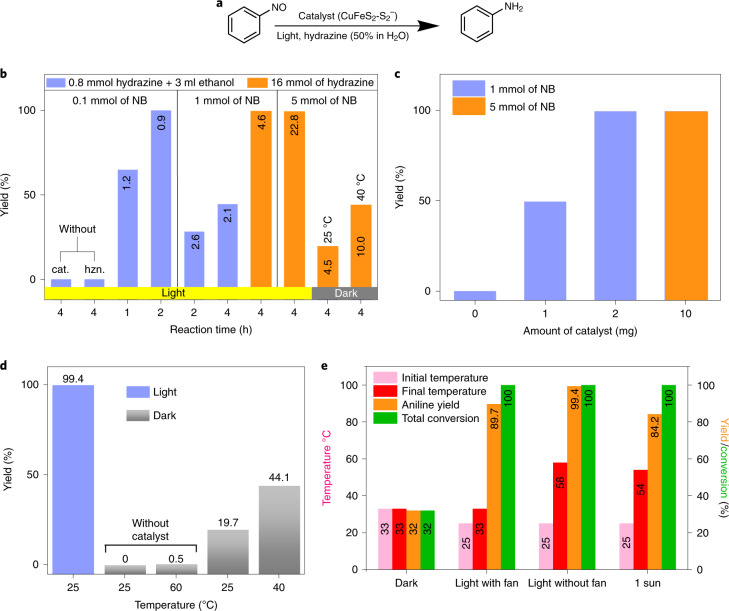
Catalytic reaction study. **a**–**e**, Reduction of nitrobenzene (NB) (**a**) using CuFeS_2_ NCs (**b**) for different reaction times and amounts of NB and hydrazine hydrate, using in all cases 10 mg catalyst (labels inside the bars are the corresponding TOF values), with different catalyst amounts (**c**) (4 h reaction time), aniline yield at different environmental temperatures (**d**) (4 h, 16 mmol hydrazine, 5 mmol nitrobenzene, 10 mg catalyst) and under controlled temperature or light (**e**). Reaction conditions for **e** were nitrobenzene, 1 mmol; hydrazine hydrate, 1 ml; catalyst, 2 mg and under light/heat irradiation with continuous stirring for 4 h. cat., catalyst; hzn., hydrazine. Source data

The photocatalytic activity of the CuFeS_2_ NCs for the hydrogenation of nitroarenes (Fig. 2a) was evaluated using hydrazine hydrate as a hydrogen and electron donor. Hydrazine is an attractive choice because of the high hydrogen content (8.0 mass%), simply separable by-products (only hydrogen and nitrogen) and scalable synthesis from ammonia. The reaction was optimized under 400–500 nm of light, at a very low flux of 22 mW cm^−2^ and maximum intensity at 450 nm. Reaction optimization using 10 mg of the CuFeS_2_ catalyst showed that at 2 h with 0.8 mmol of hydrazine afforded the product (aniline) at 100% yield and selectivity, using 0.1 mmol of the nitrobenzene substrate (Fig. 2b, left part). By increasing the amount of the substrate tenfold (1 mmol) and the amount of hydrazine to 16 mmol (in 1 ml of H_2_O) similar results were obtained at 4 h of reaction (Fig. 2b, middle part), corresponding to a molar TOF of 4.6 h^−1^, this being already among the highest reported (Supplementary Table 1; TOF is calculated with respect to the total moles of all components of the catalyst, as explained in the notes of the same Table 1). It was very gratifying to observe that by further challenging the catalyst via increasing the substrate to 5 mmol under the exact same conditions, aniline was again obtained at 100% conversion and selectivity, affording the highest TOF value of 22.8 h^−1^ (Fig. 2b, right part). Reactions without catalyst or without hydrazine did not yield any aniline, while a control reaction in the dark at 25 °C delivered a yield of 19% (Fig. 2b), suggesting intrinsic catalytic activity of the system. CuFeS_2_ NCs coated with the oleylamine molecules (Supplementary Fig. 7) showed lower yield than the S^2−^ passivated NCs. The reaction yield and rate depended on the amount of the catalyst (Fig. 2c) reaching a maximum yield of 99.4% and a molar average TOF of 22.8 h^−1^ with an optimum catalyst to substrate ratio of 10 mg per 5 mmol of nitrobenzene. This TOF is substantially higher than any recently disclosed state of the art thermal catalyst or photocatalyst for nitroarene reduction, as later discussed and described in Supplementary Table 1. Even in a large-scale reaction with 20 mmol (2.5 g) of nitrobenzene, the TOF was retained at 22.2 h^−1^ (Supplementary Figs. 8–12).

To gain further insights, control reactions were performed in the dark in an oil bath at 25 or 40 °C, affording aniline with a yield of 19.7 and 44.1%, respectively (Fig. 2d), verifying that CuFeS_2_ NCs are intrinsically active, which is an important feature of an ideal photocatalyst^32^. Control experiments were performed using Cu, Fe or S elements, as well as Fe_2_O_3_, FeCl_3_ CuI and mixtures thereof, which showed very low activity (Supplementary Fig. 7). CuFeS_2_ is also a well-known photothermal agent^29^, thus light irradiation during the catalytic reaction caused a spontaneous temperature increase reaching 58 °C (Fig. 2e, ‘light without fan’). When the same reaction was performed using the cooling fan of the photoreactor, the temperature stabilized at 33 °C, giving a slightly lower yield of 89.7% with 100% nitrobenzene conversion (Fig. 2e, ‘light with fan’). Control reactions in the dark at 40 or 33 °C delivered lower yields (44.1 and 32%, respectively, Fig. 2d,e ‘dark’) than the reaction at 33 °C but under light, indicating that the NCs did not act only through photothermal activation, but also through intermediate photoexcited species. The catalyst was finally challenged using a 1-sun solar-light simulator, delivering a TOF of 20 h^−1^ (around 84.2% yield) within 4 h (Fig. 2e, 1 sun). The slightly lower selectivities in the presence of light with fan-cooling and with the sun-simulator (Fig. 2e) are probably attributed to the lower temperature and broader irradiation spectrum, respectively.

The importance of these results can be better recognized if evaluated within the state of the art. For instance, a Zn-based metal organic framework^15^ showed very efficient nitrobenzene photo-reduction (TOF = 13.3 h^−1^, Supplementary Table 1, entry υ), using very high intensity of light and costly organic ligands (detailed description of costs is available in the Supplementary Information). A Pd_3_Au_0.5_/SiC photocatalyst showed excellent nitroaromatic hydrogenation^22^ (TOF = 7.9 h^−1^, Supplementary Table 1, entry φ), but with the need of high-cost noble metals, H_2_ flow and high fluence of light (300 mW cm^−2^ as opposed to the 22 mW cm^−2^ in the present case). Semiconductor photo-catalysts, such as Cu_*x*_S-ZnCdS (TOF = 3.9 h^−1^) and Zn_1 − *x*_Cd_*x*_S (TOF = 1.1 h^−^^1^, Supplementary Table 1, entries τ and π, respectively), also showed good activity, but the toxic heavy metals^9,17^ rise environmental concerns. Even in some exemplary cases of highly sustainable catalysts of iron and cobalt oxides embedded on nitrogen-doped graphitic layers for the efficient chemo-selective hydrogenation of nitroarenes^5,6^, harsh conditions were required, such as pressurized H_2_ (50 bar) at 110–120 °C (Supplementary Table 1, entries a,b). Moreover, single Co atoms in N-doped carbon^13^ or Co nanoparticles encapsulated in carbon nanotubes^12^ achieved high activity under relatively benign reaction conditions, but still requiring 2–4 bar of H_2_ pressure^12,13^ and temperature of 110 °C (ref. ^12^). However, the present catalyst delivered higher reaction rates while using low-cost and sustainable metals without any other energy input than the irradiation from solar light.

### Recyclability and substrate scope

**Fig. 3:**
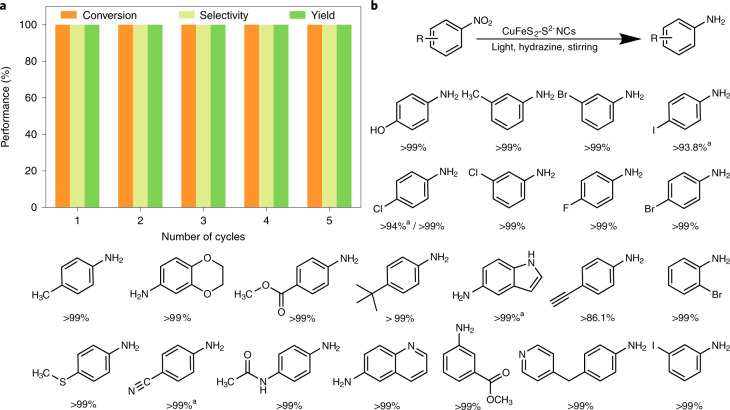
Catalyst recyclability and substrate study. **a**, Recycling performance of the catalyst for the photocatalytic reduction of nitrobenzene. Reaction conditions were 0.1 mmol nitro compound, 50 µl hydrazine hydrate, 10 mg catalyst, 3 ml ethanol and light irradiation with continuous stirring at room temperature for 4 h. **b**, Photocatalytic reduction of nitroarenes to anilines catalysed by CuFeS_2_ NCs. The percentiles correspond to the reaction yields, as determined by gas chromatography. Reaction conditions were 0.1 mmol nitro compound, 50 µl hydrazine hydrate, 10 mg catalyst, 3 ml ethanol and light irradiation with continuous stirring at room temperature for 4 h. The asterisk (^a^) denotes 1 mmol nitroarene, 1 ml hydrazine hydrate and 2 mg catalyst under light irradiation with continuous stirring at room temperature for 4 h. Source data

The recyclability of the CuFeS_2_ nano-catalyst was investigated for five consecutive reactions with 1 mmol of nitrobenzene and 2 mg of catalyst (that is, at its maximum performance, Fig. 3a and Supplementary Fig. 13). The results indicated that there was marginal loss in the catalytic activity even after the fifth cycle (100% conversion and 86% yield or better at conditions lower than its maximum performance, Supplementary Fig. 14). Moreover, there was no need for increasing the reaction time or the pressure and temperature, as often required^6,13^. XPS analysis before and after the reaction (Supplementary Fig. 5) confirmed the preservation of its structural features. Besides the high activity of the catalyst, its ability to reduce effectively a wide variety of substrates with high selectivity (Fig. 3b), irrespectively of the presence of other functionalities, is of additional importance. Challenging substrates, with competing reducible groups (that is, 4-nitrobenzonitrile, 4-iodo-nitrobenzoate and 4-ethynylnitrobenzene^5,6^) were obtained with yields of 99, 93.8 and 86.1%, respectively. Indicatively, previously achieved yields of 4-nitrobenzonitrile and 4-ethynylnitrobenzene were 75 (ref. ^5^) and 83% (ref. ^6^), respectively, at high temperature and 50 bar H_2_ atmosphere.

### Benchmarking of the catalyst

**Fig. 4:**
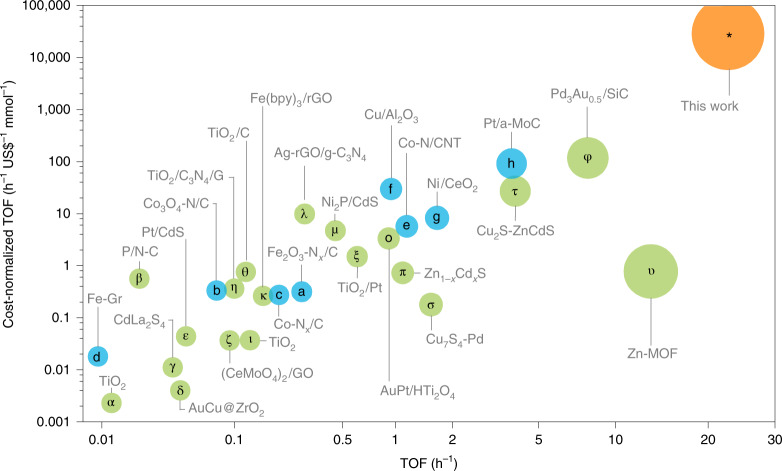
The catalyst performance with respect to the state of the art. Comparisons of the average TOF values and of the cost-normalized TOF for the CuFeS_2_ catalyst and for previously reported ones, under photocatalytic conditions (Greek alphabet letters in green) and under elevated temperature and H_2_ pressure conditions (Latin alphabet letters in blue). α, ref. ^40^; β, ref. ^41^; γ, ref. ^42^; δ, ref. ^23^; ε, ref. ^43^; ζ, ref. ^44^; η, ref. ^45^; θ, ref. ^46^; ι, ref. ^47^; κ, ref. ^14^; λ, ref. ^48^; μ, ref. ^16^, ξ, ref. ^49^; ο, ref. ^21^; π, ref. ^9^; σ, ref. ^8^; τ, ref. ^17^; υ, ref. ^15^; φ, ref. ^22^; a, ref. ^5^; b, ref. ^6^; c, ref. ^13^; d, ref. ^10^; e, ref. ^12^; f, ref. ^7^; g, ref. ^11^ and h, ref. ^50^: more details are given in Supplementary Table 1. Source data

To interpret our results within the context of the current state of the art and with respect to the related costs, we collected data on the TOF values as well as on TOF with respect to the cost of the catalyst (Supplementary Table 1 and Fig. 4). For an unambiguous comparison, we included the whole catalyst system for calculating the TOF; regarding the price, we took into account the initial key reagents used in the synthesis of the catalysts, considering 100% yield (details are given in the Supplementary Table 1, in the Experimental section in the Supplementary Information). According to this analysis, the present CuFeS_2_-S^2−^ plasmonic photocatalyst revealed its high production rate and a transformative performance based on TOF with respect to the catalyst costs (Fig. 4).

### Insights into the mechanism of action of the CuFeS_2_ plasmonic photocatalyst

**Fig. 5:**
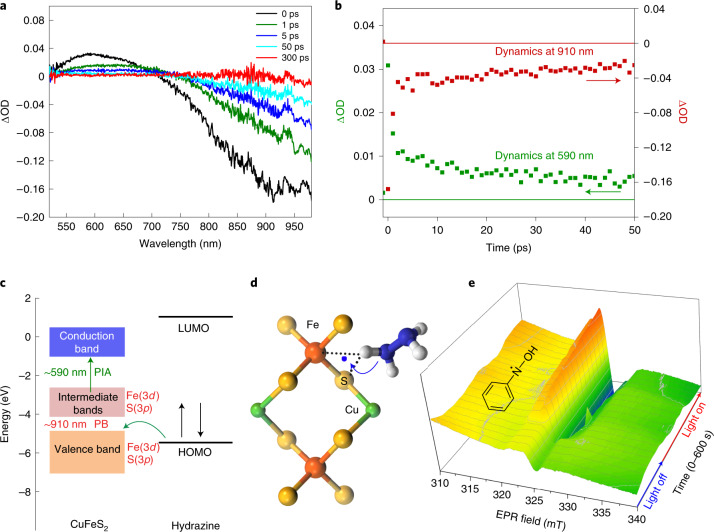
TAS and light-induced electron paramagnetic resonance studies of the catalyst. **a**, Time-resolved transient absorption spectra of the CuFeS_2_ catalyst showing the optical density difference (ΔOD) as a function of wavelength at various time delays. **b**, Transient dynamics of the CuFeS_2_ PIA at 590 nm and photobleaching (PB) at 910 nm. **c**, Schematic representation of energy level diagrams of CuFeS_2_ and hydrazine. LUMO, lowest occupied molecular orbital. **d**, The photoexcited intermediate specie of the catalyst with hydrazine, in accordance with hydrazine’s oxidation by transferring electrons from its HOMO to the energy-matching photogenerated holes in the valence band of CuFeS_2_ (**c**). **e**, The emergence of the three-electron reduction intermediate of nitrobenzene upon light irradiation. Source data

To better understand the high activity of the catalyst, ultrafast laser time-resolved transient absorption spectroscopy (TAS) and continuous-wave light-induced electron paramagnetic resonance experiments were performed (Fig. 5). In TAS studies, the difference in optical density (ΔOD) at various time delays and wavelengths (Fig. 5a,b) revealed the presence of two main processes: (1) a photo-induced absorption (PIA) and (2) a photobleaching feature in the vicinity of 590 and 750 nm, respectively. The PIA profile is attributed to transitions from temporary occupied states in the intermediate bands to the conduction band^28,29^, while the simultaneously observed photobleaching feature is attributed to transitions from the depleted valence band to states within the intermediate band^28,29^.

The decay dynamics of these two relaxation processes unveiled that both PIA and photobleaching exhibited an identical two-step decay profile, with a fast component of few ps, followed by a slower component of several tens of ps (Fig. 5b). The fast time component is related to the nonradiative intraband electron–electron and electron–phonon scattering relaxation processes taking place in the intermediate band and in the conduction band, which results in carrier cooling on transferring the excess energy of the excited electrons to the crystal lattice, ultimately leading to NC heating^20,29,30,33^. The slower time component is attributed to the heat transfer to the surrounding environment of the nanoparticles^20,29,30,33^. The very similar fast decay profiles of PIA and photobleaching features indicate that hot electrons and heat are generated in both the conduction band and the intermediate band. Although these timescales are beyond the fs processes of Landau damping (when hot electron–hole pairs are generated^33^) and therefore cannot be observed, nonradiative plasmonic nanostructures (such as CuFeS_2_) favour hot electron generation and heating^20,30,33^. According to the theoretically calculated band structure of CuFeS_2_ (ref. ^34^), the intermediate band–conduction band gap is about 2 eV (Fig. 5c), corroborating the PIA feature at the spectral window around 590 nm (2.1 eV). The valence band–intermediate band gap is 0.7–1 eV, matching the photobleaching feature with maximum around 910 nm, which was also verified by the Tauc plots, at around 0.85 eV (Supplementary Fig. 15). The full agreement between the experimental and theoretical data clearly supports the formation of holes in the valence band of CuFeS_2_ (with maximum energy of around −5.2 eV^35^) and hot electrons in the intermediate band and conduction band (at around −4 eV and above −2 eV, respectively, Fig. 5c). At the same time, the HOMO of hydrazine is positioned at −5.1 eV (ref. ^36^), extremely close to the upper valence band energy levels of CuFeS_2_, where the holes are created. This energy matching promotes a favourable interaction of hydrazine’s antibonding and bonding HOMO electrons with the holes from CuFeS_2_ valence band (generated during photoexcitation), which leads to weakening the N–H bond, proton and electron abstraction from hydrazine via formation of the intermediate complex, as depicted in the possible structure of Fig. 5d. Through continuous-wave light-induced electron paramagnetic resonance experiments, we observed such an interaction and electron transfer from hydrazine to the catalyst in water before the addition of nitrobenzene, revealing a new photoexcited spin state (Supplementary Figs. 20 and 21) with hyperfine parameters suggesting the structure of Fig. 5d (and Supplementary Fig. 21e). On the addition of nitrobenzene, a new radical species produced a strong signal as time evolved (Fig. 5e and Supplementary Figs. 22 and 23). This type of signal corresponds to *N*-phenylhydroxylamine radical species (–N^•^–OH), as verified by the simulated spectrum with the corresponding spin-Hamiltonian parameters (Supplementary Fig. 23d) and by the spin-trap experiments (Supplementary Fig. 24). This radical can be associated with the three-electron reduced intermediate form of nitrobenzene (highlighted in the reaction mechanism in Supplementary Fig. 27), identifying a possible and previously elusive three-electron intermediate in the overall reaction pathway A. Further support for the predominance of pathway A in the presence of light is provided by gas chromatography results, showing hydroxylamine or azoxybenzine as the only stable intermediates in the presence of light or in the dark, respectively (Supplementary Fig. 28). The energy matching of the catalyst’s photogenerated holes with the HOMO of hydrazine could be considered responsible for the excellent performance of the catalyst.

CuFeS_2_ NCs also use the synergic contribution of the two metal centres, Fe and Cu. The Fe site is responsible for binding and activating hydrazine, forming the transient spin‑active species, [H(FeS_2_)NH-NH_2_]^•^, *S* = 1/2 system, which delivers the protons and electrons to the neighbouring Cu(I)S_2_ site. The Cu(I)S_2_ sites interact with the nitro-substrate, producing the *N*-phenylhydroxylamine radical, as experimentally trapped in situ (Supplementary Fig. 23). The results take forward the concept that by a judicious combination of metal centres bound to rigid ligand-field environments, a highly effective catalytic system can be conveyed, harnessing the power of cooperative enzymatic catalytic centres^37^, for example, to effectively transfer H^+^ and e^−^ to the substrate^38^. The use of the identified energy flow pair (CuFeS_2_-H_2_NNH_2_) extends beyond this reaction, affecting a broad family of hydrogen transfer and reduction catalytic reactions in valuable processes for biomass valorization^39^.

## Conclusions

A highly efficient heterogeneous plasmonic photocatalyst is developed for the important catalytic reduction of nitroaromatics into amines, based on nontoxic and earth-abundant chalcopyrite NCs. The catalyst can spontaneously raise the reaction temperature and form photoexcited intermediate complexes with the reactants, delivering particularly high reaction rates even for demanding substrates with sensitive side-groups, as well as after its recycling under conditions of its maximum production rate. The production rate of this catalytic system is higher compared to other top-rated photo and thermal catalysts, with its cost-normalized rate standing an order of magnitude above the current state of the art. Surface modification of the catalyst with metal ions to tailor the energy of surface states and match the frontier orbitals of other substrates might further expand the importance and use of the present findings.

## Methods

Supplementary Information files contain detailed descriptions of the methods used in this study.

## Online content

Any methods, additional references, Nature Research reporting summaries, source data, extended data, supplementary information, acknowledgements, peer review information; details of author contributions and competing interests; and statements of data and code availability are available at 10.1038/s41565-022-01087-3.

## Supplementary information

Supplementary Information Supplementary Material and methods, Figs. 1–24 and Table 1.

Supplementary Data 1 The file contains the detailed description of the chemicals and costs as obtained from links from the providers for all the reports from literature that were used for comparisons.

## Data Availability

All data that support the findings of this study are available in the main text, figures and Supplementary Information files. Further data enquiries can be addressed to the corresponding authors on reasonable request. Source data are provided with this paper.

## Source data

Source Data Fig. 1 Raw data from the instruments (EDS, absorption, XRD and FTIR).
Source Data Fig. 2 Gas chromatography yield values used to plot the graphs.
Source Data Fig. 3 Gas chromatography yield, conversion and selectivity values used to plot the graph.
Source Data Fig. 4 Calculated TOF values and cost used to plot the graph.
Source Data Fig. 5 Raw data from the instrument (TAS and electron paramagnetic resonance).
