# Supplementary material for: Fast and selective reduction of nitroarenes under visible light with an earth-abundant plasmonic photocatalyst
Source: Nat Nanotechnol. 2022 Mar 28;17(5):485–92. doi: 10.1038/s41565-022-01087-3 (PMC9117130; doi:10.1038/s41565-022-01087-3)
Supplement: Supplementary file 1 — Supplementary Material and methods, Figs. 1–24 and Table 1. [file 41565_2022_1087_MOESM1_ESM.pdf]

nature portfolio

https://doi.org/10.1038/s41565-022-01087-3

Supplementary information

Fast and selective reduction of nitroarenes under visible light with an earth-abundant plasmonic photocatalyst

In the format provided by the authors and unedited

Supplementary Information

Fast and Selective Reduction of Nitroarenes Under Visible Light with an Earth-Abundant Plasmonic Photocatalyst

Aby Cheruvathoor Poulose,[1] Giorgio Zoppellaro,[1] Ioannis Konidakis,[3] Efthymis Serpetzoglou,[3] Emmanuel Stratakis,[3] Ondřej Tomanec,[1] Matthias Beller,[4] Aristides Bakandritsos,[1,2,*] Radek Zboril[1,2,*]

[1]Regional Centre of Advanced Technologies and Materials, Czech Advanced Technology and Research Institute, Slechtitelu 27, 77900, Palacký University, Olomouc, Czech Republic

[2]Nanotechnology Centre, Centre of Energy and Environmental Technologies, VŠB–Technical University of Ostrava, 17. listopadu 2172/15, 708 00 Ostrava-Poruba, Czech Republic

[3]Institute of Electronic Structure and Laser Foundation for Research & Technology – Hellas 711 10 Heraklion, Greece

[4]Leibniz-Institute for Catalysis, Albert-Einstein-Str. 29a, 18059 Rostock, Germany

* Corresponding authors E-mail addresses: a.bakandritsos@upol.cz, arisbakan@gmail.com; radek.zboril@upol.cz

1. Materials and methods

Synthesis of $CuFeS_2$ NCs. In a typical synthesis, 1 mmol (0.192 g) of CuI and 1 mmol (0.162 g) of $FeCl_3$ were dissolved in 27 mL of oleylamine and stirred in a three-necked round bottom flask. The mixture was heated to 100 °C under vacuum and maintained for 2 h to remove oxygen and moisture. The reaction atmosphere was then switched to nitrogen and then raised to 180 °C and stirred until the solution obtained a clear orange color. 2 mmol of hexamethyldisilathiane (or 2 mmol of sulfur powder) dissolved into 3 mL of 1-octadecene (ODE) was swiftly injected into the above mentioned mixture under vigorous stirring, and the temperature was kept for 10 min at 180 °C for the growth of the $CuFeS_2$ NCs. Then, the solution was cooled to room temperature under ambient conditions. Toluene and ethanol were added to the reaction mixture for at 9418 rcf for 5 min. The precipitate was then washed twice with an 1:1 ethanol:toluene mixture for two times. Finally, the oleylamine-capped nanocrystals were collected and dried at 60 °C under reduced pressure.

1.2. Ligand exchange of the $CuFeS_2$ NCs

For exchanging the oleylamine capping ligands of the $CuFeS_2$ nanocrystals a procedure was adapted from prior literature with slight modifications[1,2]. 0.1 g of $Na_2S.9H_2O$ was dissolved in 5 mL of ultrapure water and then 15 mL of dimethylformamide was added, to form the sulfide ligand-exchange solution. 0.2 g of $CuFeS_2$ NCs were dispersed in 10 mL cyclohexane and then mixed with ligand-exchange solution and stirred for 30 min at room temperature. The mixture was centrifuged at 9418 rcf for 5 min and the supernatant was discarded. The precipitate was washed 3 times with ethanol and the ligand-exchanged nanocrystals were dried under vacuum at 60 °C. The final solid catalyst was grinded well in a mortar with a pestle before use.

1.3. Characterization

Transmission electron microscopy (TEM) images were recorded on JEOL JEM-2100 TEM equipped with a LaB_6 type emission gun operating at 200 kV. High-resolution transmission electron microscopy (HR-TEM) and scanning transmission electron microscopy (STEM) in high-angle angular dark field (HAADF) mode for elemental mapping were performed with an FEI TITAN G2 60-300 HRTEM microscope with an X-FEG type emission gun, operating at 300 kV, objective-lens image spherical aberration corrector and ChemiSTEM EDS detector. X-ray diffraction (XRD) patterns were recorded with a PANalytical X'Pert PRO MPD (PANalytical, The Netherlands) diffractometer in the Bragg-Brentano geometry, Co-Kα radiation (40 kV, 30 mA, λ = 0.1789 nm) equipped with an X'Celerator detector and programmable divergence and diffracted beam antiscatter slits. The measurement range was 2θ:0 5°–105°, with a step size of 0.033°. The identification of the crystalline phases was performed using the High Score Plus software (PANalytical) that includes the PDF-4+ database. X-ray photoelectron spectroscopy (XPS) was carried out with a PHI VersaProbe II (Physical Electronics) spectrometer using an Al Kα source (15 kV, 50 W). The obtained data were evaluated with the MultiPak (Ulvac - PHI, Inc.) software package. UV–Vis absorption spectra were collected on a Cary 50 UV–Vis spectrophotometer (Varian). Fourier transform infra-red (FT-IR) spectra were recorded on an iS5 FTIR spectrometer (Thermo Nicolet) using the Smart Orbit ZnSe ATR accessory. Briefly, a droplet of chloroform/ethanol dispersion of the relevant material was placed on a ZnSe crystal and left to dry and form a film. Spectra were acquired by summing 64 scans recorded under a nitrogen gas flow through the ATR accessory. ATR and baseline correction were applied to the collected spectra. Raman spectra were recorded on a DXR Raman microscope using the 613 nm excitation line of a diode laser

EPR spectra were recorded on a JEOL JES-X-320 spectrometer operating at the X-band frequency (~9.0–9.1 GHz) equipped with a variable-temperature controller (He, N_2) ES-CT470 apparatus. The cavity quality factor (Q) was kept above 6000 in all measurements. Highly pure quartz tubes were employed (Suprasil, Wilmad, ≤0.5 OD), and accuracy on g-values was obtained against the Mn^{II}/MgO standard (JEOL standard). The spectra were acquired by careful monitoring that signal saturation from the applied microwave power did not occur during signal's acquisition. *In* situ light excitations EPR experiments (LEPR) were performed using a HeCd laser source operating @325 nm (max CW power of 200 mW) from Kimmon Koha Co. Ltd. The light was shined directly onto the sample, kept inside the cavity EPR resonator, through its dedicated optical window and the light-off to light-on process was operated by an on-off mechanical light-shutter mechanism.

The band gap of the $CuFeS_2$ NCs was calculated using the Tauc equation and was found to be 0.85 eV for the direct band gap and 1.72 eV for the indirect band gap, corresponding to transitions from the valence band to the intermediate band and from the intermediate band to the conduction band, respectively. A Lambda 1050 UV/Vis/NIR spectrophotometer (PerkinElmer) was used.

1.4. Catalytic nitrobenzene reduction

2 and up to 10 mg of $CuFeS_2$ catalyst was added in a glass vial with a teflon-coated cap (5 mL), adding 0.1 and up to 5 mmol of nitrobenzene, 0.05 mL to 1 mL of hydrazine hydrate (50 % solution) and 0 to 3 mL of ethanol. After mixing by stirring (60 s) and sonication (30 s), the closed vial was irradiated in a photoreactor equipped with a light source (EvoluChem PhotoRedOx box, attached with 34 W Kessil 150N LED having total irradiance of 22 mW/cm^2 from 400-500 nm region, peaking at 450 nm) for 0 to 4 h under stirring at room temperature.

For a larger-scale reaction, 40 mg of catalyst and 4 mL of hydrazine hydrate were mixed well and sonicated for 30 s in a closed reaction vessel (60 mL capacity), and 20 mmol (2.46 g, 2 mL) of

nitrobenzene was added. The reaction vessel was irradiated in the same photoreactor chamber for 4 hours under stirring. After the reaction, the products were extracted by ethyl acetate and the catalyst separated by centrifugation. The ethyl acetate solution of the product was further diluted and analyzed in GC and NMR.

Reactions were also performed using a solar simulator of 1 Sun (100 $mWcm^2$) intensity (Sciencetech Light Line A4-C250 equipped with an AM 1.5G filter, class CAA). After the reaction, the catalyst was separated by centrifugation and the product was obtained in ethanol or ethyl acetate and analyzed by GC (Agilent 6820, United States), equipped with flame ionization detector (FID). Products were identified by comparison of the retention time with standard chemicals and the quantitative analysis of the each content was analyzed by GC *via* interpolation from calibration curves.

Equations used to calculate the conversion, yield and selectivity are given below.

$$\text{Conversion (\%)} = \frac{\text{Moles all products}}{\text{Moles of reactant}} \times 100$$

$$\text{Yield (\%)} = \frac{\text{Moles of desired product}}{\text{Moles of reactant}} \times 100$$

$$\text{Selectivity (\%)} = \frac{\text{Moles of desired product}}{\text{Moles of all products}} \times 100$$

Turnover frequency (TOF) was calculated according to the following equation:

$$\text{TOF} = \frac{\text{Moles of desired product}}{\text{Moles of catalyst} \times \text{hours of reaction}}$$

The moles of the catalyst were calculated by considering the dry weight and molecular formula ($CuFeS_2$) of the catalyst. For example, 10 mg of the $CuFeS_2$ catalyst was equal to 54.49 µmol, considering 183.52 as molecular weight.

As an example, the recalculation method of the TOF value for entry σ ($Pd_3Au_{0.5}$/SiC; ref 21) is also described. In the article a TOF value of 900 h^{-1} is reported based on the metal content only, at

300 mW/cm^2 of irradiation intensity. However, here, we consider the whole catalyst system for calculating the TOF, since the support is an integral part of the catalyst defining its catalytic activity (*e.g.* with different supports the catalyst displayed different activity; actually, certain conditions SiC is catalytically active, Fig. S16 ref 21). The total catalyst used for converting 6 mmol of nitroarene was 25 mg (609.15 µmol), from which 24.125 mg (601.47 µmol) corresponded to SiC, 0.125 mg (0.63 µmol) to Au and 0.75 mg (7.04 µmol) Pd. Therefore, the molar-based TOF value was equal to 7.9 h^{-1} (for 6 mmol reactant, 609.15 µmol catalyst, 0.5 h reaction time, and 80 % product yield).

The calculations for the cost-normalized TOF values (TOF \$$^{-1}$ mmol) were performed considering the commercially available precursors for the synthesis of the catalyst and that the catalyst was obtained at 100 % yield. Metal precursors and other reagents necessary for the synthesis of the catalyst were considered, except common solvents. In all cases, the cost of the catalyst corresponding to the mass used for the conversion of 1 mmol of substrate was considered:

$$\text{Cost-normalized TOF} = \frac{\text{TOF } (h^{-1})}{\text{cost of catalyst per 1 mmol of product } (\$ \times \text{mmol}^{-1})}, h^{-1}\ \$^{-1}\ mmol$$

A detailed excel file, where all the cost calculations are described in detail, is available as a Supplementary data file.

1.5. Recyclability and substrate study

The recyclability of the catalyst was tested in the reaction of 1 mmol of nitrobenzene, in 1 mL of hydrazine hydrate, using 2 mg of the catalyst. The above regents were mixed well and sonicated for 30 s in a closed reaction vessel and then irradiated with for 4 hours under stirring at room temperature. After the reaction, the products were extracted by ethyl acetate and the catalyst separated by centrifugation. The ethyl acetate solution of the product was further diluted and analyzed in GC. The recovered catalyst was washed several times with ethanol and

dichloromethane to remove any adsorbed molecules and finally dried under vacuum oven overnight at 60 °C before using it for the next cycle.

1.6. Transient absorption spectroscopy (TAS) study

Transient absorption spectroscopy (TAS) measurements were performed on a Newport (TAS-1) transient absorption spectrometer, equipped with a source pulsed laser beam generated from an Yb:KGW-based laser system (PHAROS, Light Conversion), emitting at 1026 nm, with a pulse duration of 170 fs and 1 KHz repetition rate[3-5]. As shown schematically in Supplementary Fig. 1 the 1026 nm fundamental beam was split, so that the probe beam component (10 % of the source) passes through a delay line and routed on a YAG crystal, which generates a supercontinuum white light of 550-920 nm. The other part of the 1026 nm incident beam (90 % of the source) was used as the pump beam for sample excitation. The energy of the pump beam was controlled by a variable reflective neutral density filter inside the TAS instrument. The probe light was coupled through an optical fiber to a multichannel detector and monitored as a function of wavelength. In a typical TAS pump-probe experiment, the sample is excited by the pump beam, and the corresponding decay dynamics of the sample's relative optical density are recorded as a function of wavelength at various time delays after photo-excitation. In the present study, the employed pump fluence of the $CuFeS_2$/toluene solution was 1.5 mJ cm^{-2}, while a delay time resolution of 1 ps was used.

2. Supplementary Tables and Figures

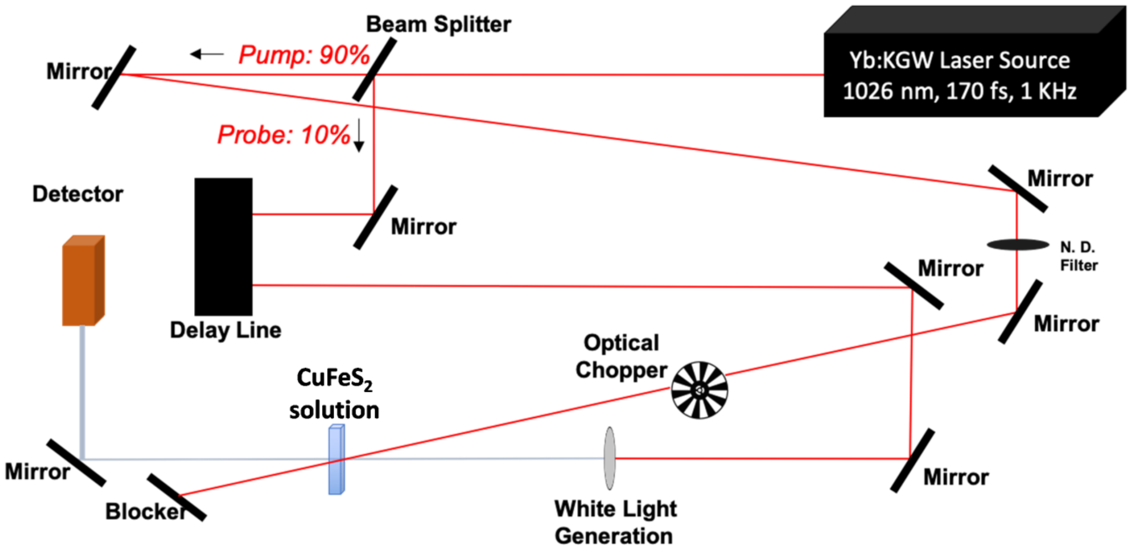

Supplementary Fig. 1: Schematic representation of transient absorption spectroscopy (TAS) experimental setup (Newport, TAS-1).

Supplementary Table 1. Comparison of the $CuFeS_2$ NCs in terms of TOF and cost for photocatalytic aromatic nitro-reduction reactions with state-of-the-art catalysts.

	Entry /Ref	Reaction type [a]	Substrate concentration	Hydrogen donor/ hole scavengers	Catalyst / amount	Yield (%)	Reaction time (h)	TOF (h^{-1})	Space-Time Yield[b] (h^{-1})	Specific productivity[c] (mmol g^{-1} h^{-1})	TOF/ $/mmol[d]	Reference
**Photo catalysts**	α	400 W	Nitrobenzene (30μmol)	methylamine (100 μmol)	TiO_2 (50 mg)	99	4	0.01	0.02	0.15	<0.1	[6]Appl Cat B 2020
	β	300 W	Nitrobenzene (100μmol)	hydrazine (10 mmol in 1 mL H_2O)	P/N co-doped C (10 mg)	100	6	0.02	0.2	1.6	0.56	[7]App Surf Sci 2019
	γ	300 W	Nitrobenzene (125μmol)	benzotrifluoride (15 mL)	$CdLa_2S_4$ (100 mg)	55.9	10	0.03	0.01	0.07	<0.1	[8]App Cat B 2018
	δ	500 mW/cm^2	Nitrobenzene (100μmol)	KOH (0.1M)/IPA	$Au_{2.6}Cu_{0.4}@ZrO_2$ (50 mg)	100	6	0.04	0.04	0.33	<0.1	[9]ACS Cat 2016
	ε	300 W	Nitrobenzene (125μmol)	benzotrifluoride (15 mL)	0.03%Pt/CdS (100 mg)	70	4	0.04	0.03	0.2	<0.1	[10]J Haz Mat 2018
	ζ	500 W	Antibiotic CAP (2mg)	H_2O 100 mL	$(CeMoO_4)_2$ /GO (50 mg)	100	0.8	0.09	0.05	0.15	<0.1	[11]ACS Appl Mater Inter 2017
	η	300 W	Nitrobenzene (0.5mmol)	methanol (50 mL)	TiO_2/C_3N_4/G (100 mg)	97	4	0.10	0.15	1.2	0.36	[12]Appl Cat B 2017
	θ	300 W	Nitrobenzene (80μmol)	Pr-OH (4 mL)	TiO_2/C (10mg)	100	6	0.12	0.16	1.3	0.76	[13]ACS Sus Chem 2019
	ι	125 W	Nitrobenzene (40μmol)	H_2O:MeOH (100 mL)	TiO_2 (50 mg)	100	0.5	0.12	0.2	1.6	<0.1	[14]Sci Rep 2017
	κ	20 W	Nitrobenzene (100μmol)	hydrazine (1 mmol) /CH_3CN	$Fe(bpy)_3$/rGO (25 mg)	88	12	0.15	0.04	0.3	0.26	[15]Appl Surf Sci 2016
	λ	75 W/m^2	Nitrobenzene (1mmol)	methanol (10 mL)	Ag-rGO/g-C_3N_4 (50 mg)	100	4	0.29	0.62	5	9.84	[16]J Haz Mat 2020
	μ	90W	Nitrobenzene(150 μmol)	Na_2S/Na_2SO_3/5 mL H_2O	Ni_2P/CdS (2 mg)	100	24	0.45	0.38	31.2	4.69	[17]Chem Comm 2015, 51
	ξ	40 mW/cm^2	2,4,6 TNT (3.5μmol)	CH_3CN/H_2O (0.7/1 mL)	TiO_2/Pt (420 μg)	27	1	0.61	0.51	2.25	1.49	[18]ACS Appl Mat 2018
	ο	300 W	Cl-Nitrobenzene (0.5mmol)	$HCOONH_4$ (3.5 mmol)	AuPt/HTi_2O_4 (20 mg)	99	4	0.92	0.97	6.2	3.32	[19]ACS Catal 2018
	π	400W	Nitrobenzene (0.2mmol)	$Na_2S/Na_2SO_3/H_2O$	$Zn_{1-x}Cd_xS$ (5 mg)	99	4	1.1	1.2	10	0.73	[20]ACS Sus Chem 2017
	σ	450 mW/cm^2	Nitrobenzene (100μmol)	0.5 mL H_2O/ 1 bar H_2	Cu_7S_4-Pd (50 mg)	100	0.5	1.55	0.5	4	0.18	[21]Nano Lett 2015
	τ	300W	Nitrobenzene (0.5mmol)	$Na_2S/Na_2SO_3/H_2O$	Cu_xS-ZnCdS (20 mg)	100	1.8	3.9	1.7	13.8	27.0	[22]Nano Res 2018
	υ	300 W	Nitrobenzene (100μmol)	hydrazine (0.1 mmol in 1 mL EtOH)	Zn-MOF (5 mg)	100	4	13.3	0.62	5	0.78	[23]J Mat Chem A 2019
	φ	300 mW/cm^2	Nitroarenes (6mmol)	H_2 atm. flow	$Pd_3Au_{0.5}$/SiC (25 mg)	80	0.5	7.9	25	200	117	[24]ACS Appl Mat 2018
	**34 W 22 mW/cm^2**		**Nitrobenzene (1mmol)**	**Hydrazine, 16mmol in 1 mL H_2O**	**$CuFeS_2$ (2 mg)**	**99.4**	**4**	**22.8**	15.4	125	**28656**	**This work**
**Thermal catalysts**	a	120 °C	Nitrobenzene (0.5 mmol)	H_2 (50 bar)	Fe_2O_3-N/C (42 mg) (0.119 mmol Fe)	98	15	0.28 (1.58)*	0.1	2.9	0.32 (1.78)	[25]Science 2013
	b	110 °C	Nitrobenzene (0.5 mmol)	H_2 (50 bar)	Co_3O_4-N/C (42 mg) (0.0044 mmol Co)	99	4	0.1 (27)*	0.36	2.9	0.33 (89)	[26]Nat Chem 2013
	c	110 °C	Nitrobenzene (1 mmol)	H_2 (3.5 bar)	Co-N_x/C (40 mg) (0.0017 mmol Co)	99	1.5	0.20 (392)*	2	6.2	0.27 (536)	[27]Sci Adv 2017
	d	70 °C	Nitrobenzene (0.1 mmol)	hydrazine (0.8mmol in 1 mL EtOH)	Fe-graphene (10 mg)	99	3	0.01	0.4	3.3	<0.1	[28]Chem Eng J.2020
	e	25 °C	Nitrobenzene (1 mmol)	H_2 (20 bar)	Co-N/CNT (20 mg)	100	6	1.15	1	8.3	5.7	[29]Adv Mat 2019
	f	140 °C	Nitrobenzene (0.5 mmol)	H_2 (20 bar)	Cu/Al_2O_3 (40 mg)	92	48	0.95	0.03	0.2	29.6	[30]ACS Catal 2019
	g	210 °C	Nitrobenzene (2.4 mmol)	H_2 (20 bar)	Ni/CeO_2 (30 mg)	83.5	7	1.67	1.2	9.5	8.26	[31]ACS Appl Mat. Int. 2018
	h	35 °C	Nitrobenzene (1 mmol)	H_2 (60 bar)	Pt/a-MoC (25 mg)	99.9	1.5	3.8 (3500)*	3.28	26.6	90.97	[32]*Nat Nanotech* 2019

Notes: [a] Intensity of light in mW/cm^2 (the power is given in watts where intensity was not specified in the reference).

[b]Space time yield: the mass of product per unit time over the total mass of catalyst.

[c]Specific productivity (mass-normalised rate of product formation): the mmol of product per unit time relative to the total mass of catalyst.

[d]Cost calculations for the catalysts were performed considering the commercially available precursors for the synthesis of the catalyst and that the catalyst was obtained at 100 % yield. The metal precursors and other reagents necessary for the synthesis of the catalyst were considered, except from the solvents. An excel file, where all the cost calculations are described in detail is available as a Supplementary data file.

*TOF values reported in the original paper, based only on metal content;

Comments: Many metal catalysts are active or structurally attainable and stable only within the frame of the support where they are embedded. For example:

-Entries *a,b* refer to nanoparticles embedded or wrapped by N-doped graphitic layers.

-Entry *c* refers to single metal atoms embedded in N-doped carbons

-Entry *e* refers to nanoparticles encapsulated in carbon nanotubes

-Entry *η* refers to the Zn atoms embedded in metal organic framework

-Entry *σ* refers to Pd/Au nanoparticles whose catalytic activity strongly depends on the type of the support.

-Entry *h* refers to 0.25 % Pt/a-MoC single Pt atom catalyst, where the MoC matrix played an active and independant catalytic role for the dissociation of the nitrogen-oxygen bonds in nitrobenzene.

-Entry *φ* refers to $Pd_3Au_{0.5}$/SiC, where under certain conditions SiC is catalytically active (Fig. S16 in the respective article). The catalytic activity was highly dependent on the light intensity; at 300 mW/cm^2 it was half than that at 800 W/cm^2 (Fig. S8 in the respective article).

In these examples, the metal centers are structurally attainable, stable, or active only within the frame of their support. For this reason, in order to obtain an unambiguous calculation of the TOF, the mass/moles of the whole system was considered. In the case of nanoparticulate catalysts, only the surface atoms are catalytically active and all the inner materials act as support. The calculation of TOF based only on surface atoms (i.e. a 1 Å periphery) would result in a sharp increase of the TOF. For example, in a 9-nm nanoparticle (as the present $CuFeS_2$), a few-atom-thick exposed surface of thickness ~1 Å occupies 30 times less volume and mass than the whole particle. **Thus, the TOF in the present system would be close to a value of 700 h^{-1}.**

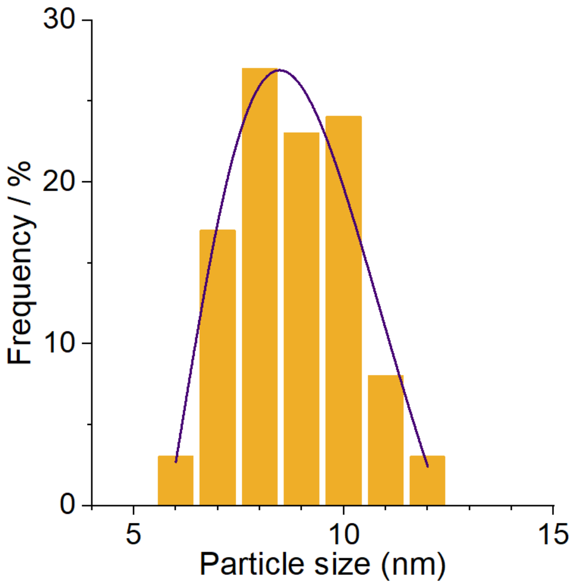

Supplementary Fig. 2: Particle size distribution of $CuFeS_2$ NCs based on the TEM.

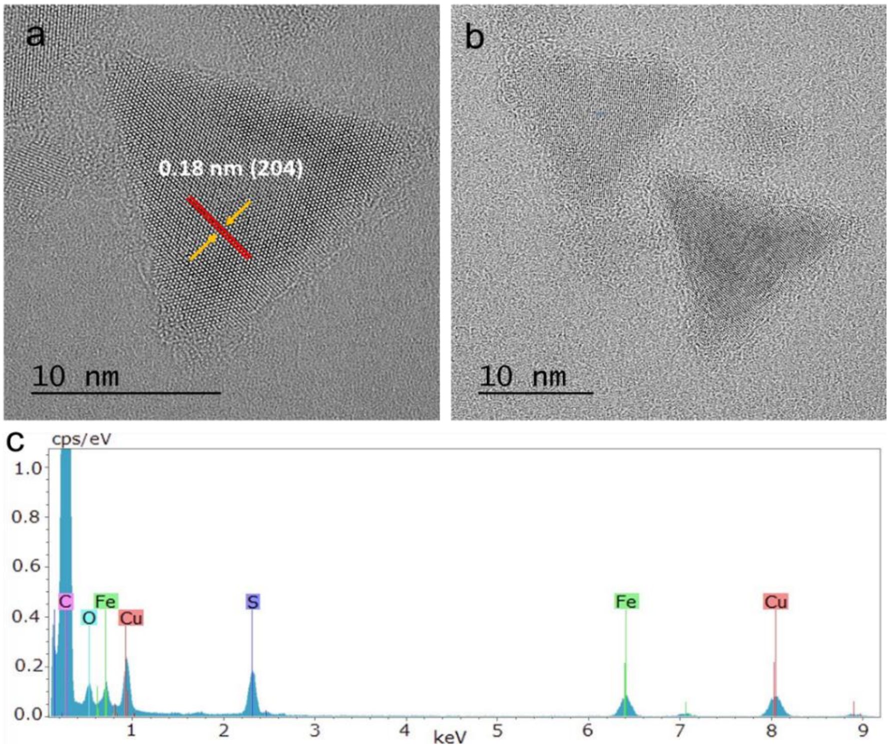

Supplementary Fig. 3: (a,b) HR-TEM images of the $CuFeS_2$ NCs. **(c)** EDS spectrum of the $CuFeS_2$ NCs.

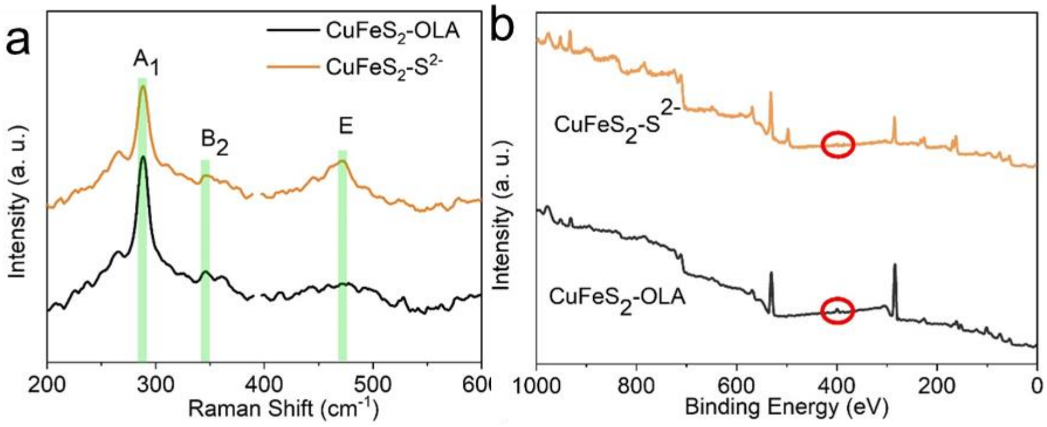

Supplementary Fig. 4: (a) Raman spectrum exhibiting the three characteristic bands of $CuFeS_2$ crystals, at 287, 351 and 470 cm^{-1}, assigned to the A_1, B_2 and E phonon modes of the $CuFeS_2$ lattice[33,34]. **(b)** XPS survey spectrum of the $CuFeS_2$ NCs, before ($CuFeS_2$-OLA) and after ($CuFeS_2$-S^{2-}) ligand exchange.

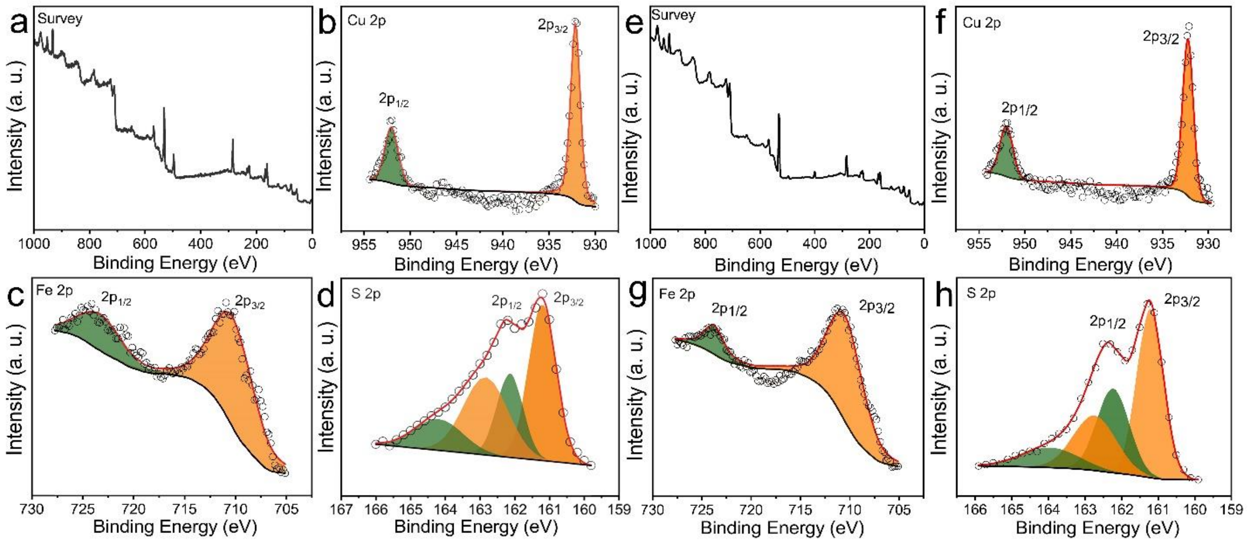

Supplementary Fig. 5: XPS spectra of the catalyst before (panels **a-d**) and after (panels **e-h**) the nitrobenzene reduction reaction.

Comments: The surface chemical states of the $CuFeS_2$ NCs before (Supplementary Fig. 5a-d) and after (Supplementary Fig. 5e-h) the reaction were also probed with X-ray photoelectron spectroscopy. The Cu 2p high-resolution XPS spectra before (Supplementary Fig. 5b) and after (Supplementary Fig. 5f) the reaction were identical, reflecting the typical spin-orbit splitting of the Cu atoms, resulting in Cu $2p_{3/2}$ and Cu $2p_{1/2}$ peaks with a separation of 19.8 eV, and no satellite peak, confirming the Cu (I) oxidation state of Cu and absence of Cu (II) species. The Fe

2p spectrum (Supplementary Fig. 5c&g) also showed the characteristic doublet due to spin-orbit splitting. The Fe $2p_{3/2}$ envelope centered at 710.9 eV, corresponding to Fe (III) oxidation state, in accordance with previous results[35]. The S 2p core level spectrum (Supplementary Fig. 5d&h) showed a spin orbit splitting (two main doublets), S $2p_{3/2}$ and S $2p_{1/2}$, corresponding to the metal-sulfide (sulfide and disulfide) bonding states of sulfur.

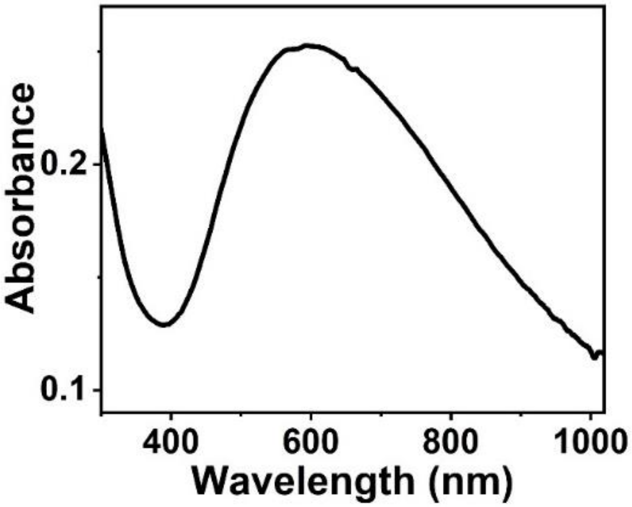

Supplementary Fig. 6: UV-Vis absorption spectra of $CuFeS_2$ NCs catalyst after nitrobenzene reduction reaction.

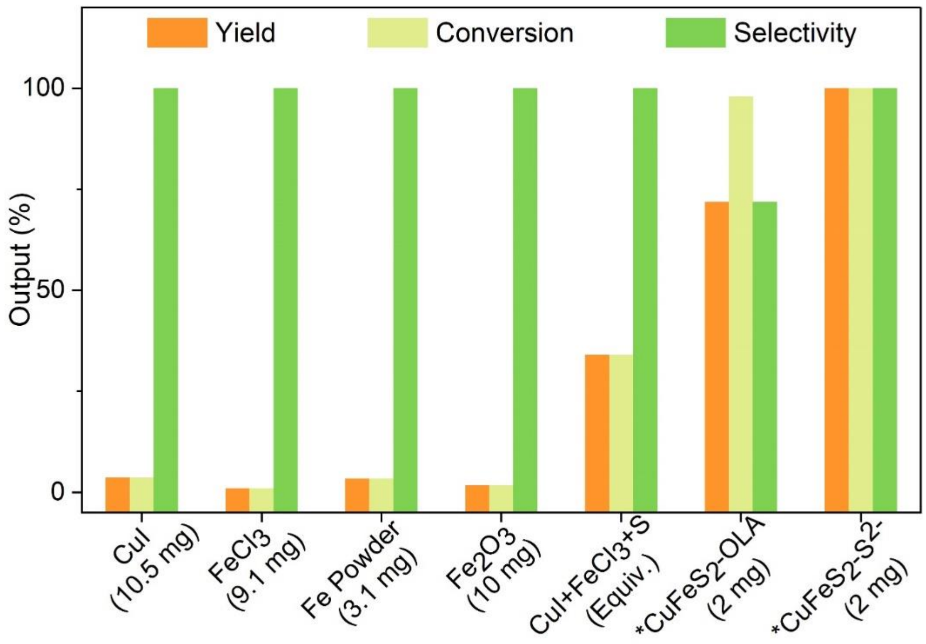

Supplementary Fig. 7: Nitrobenzene reduction with different catalysts (Reaction conditions: 0.1 mmol of nitrobenzene, 50 µl of hydrazine hydrate, corresponding amount of catalyst, 3 mL of ethanol, under light irradiation with continuous stirring at room temperature for 4 h.*same conditions with 1 mmol nitrobenzene, 1 mL hydrazine hydrate).

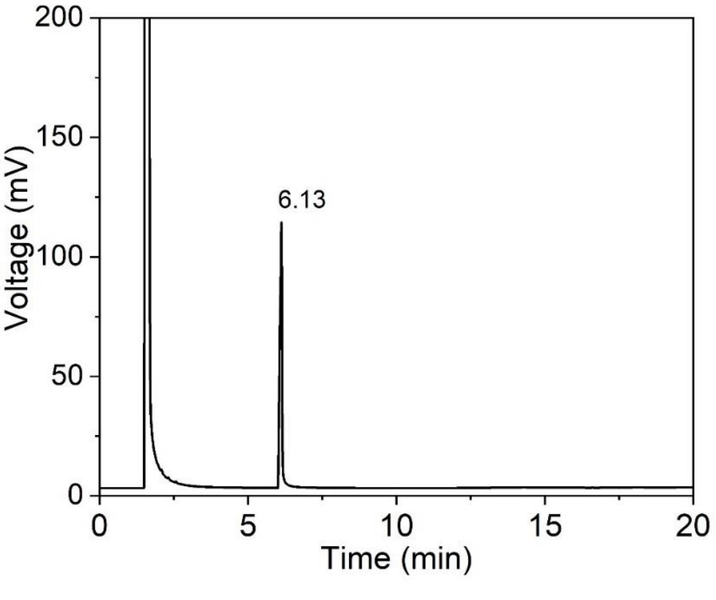

Supplementary Fig. 8: GC graph of product (aniline-6.13) produced after complete conversion of nitrobenzene (0.1 mmol) with 0.8 mmol hydrazine and 10 mg catalyst.

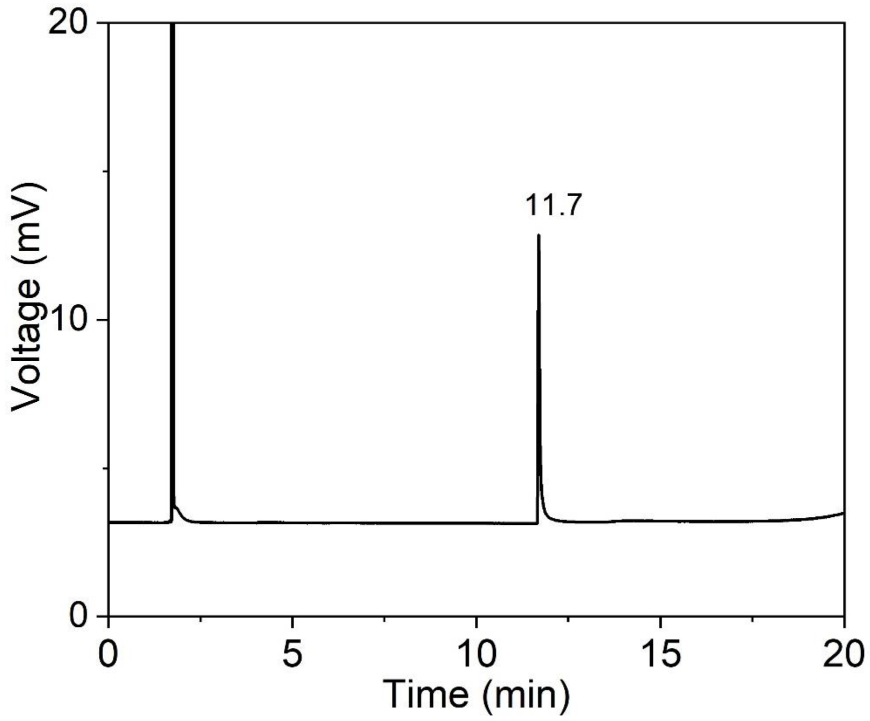

Supplementary Fig. 9: GC graph of nitrobenzene (11.22) before reaction

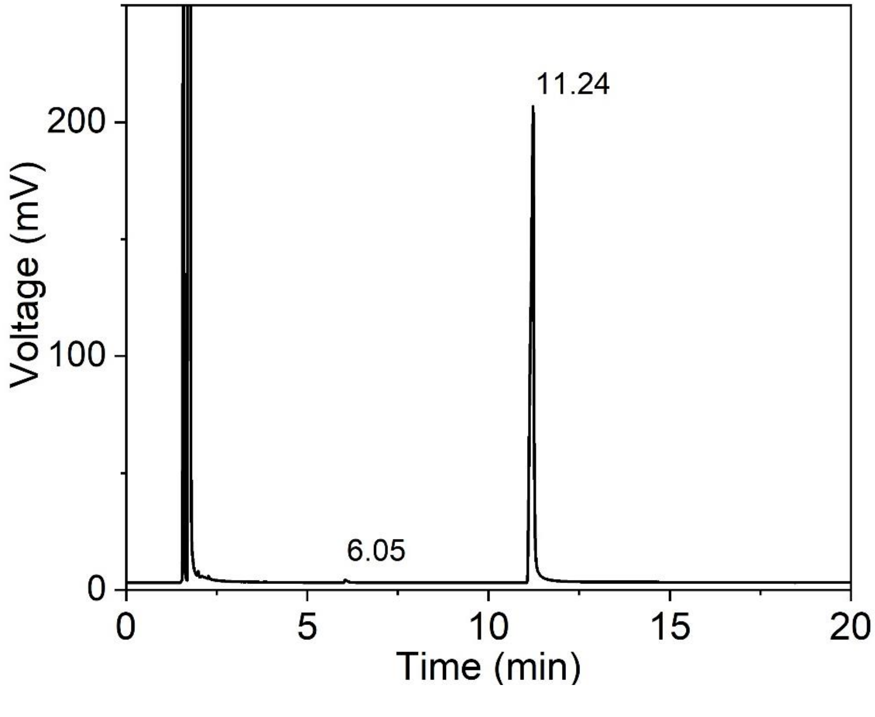

Supplementary Fig. 10: GC graph of nitrobenzene (5 mmol) reduction using catalyst (10 mg) without hydrazine. A major peak of nitrobenzene (11.61 min) and a minor product peak (6.05 min) were visible.

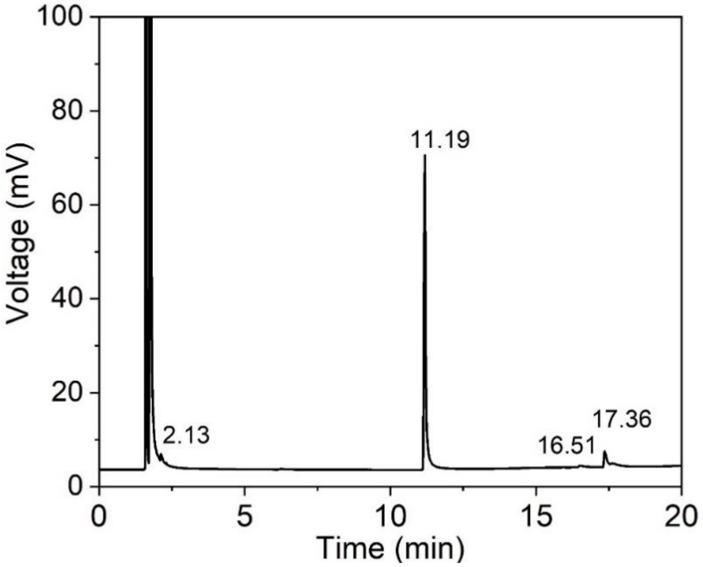

Supplementary Fig. 11: GC graph of nitrobenzene (5 mmol) reduction using hydrazine (16 mmol) without catalyst. A major peak of nitrobenzene (11.19 min) and a minor side products were visible.

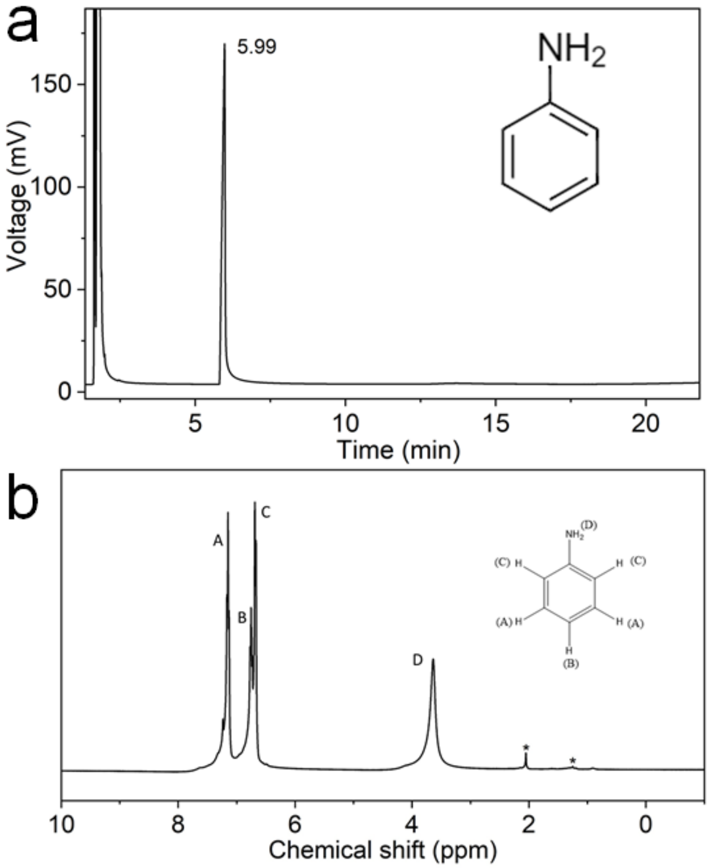

Supplementary Fig. 12: **(a)** GC (a) and **(b)** NMR spectra of the product (aniline) after the 20 mmol-scale (2.5 g) reaction. All the peaks are in agreement with the Spectral Database for Organic Compounds (SDBS) No. 905 HSP-03-391. **Solvent peaks*.

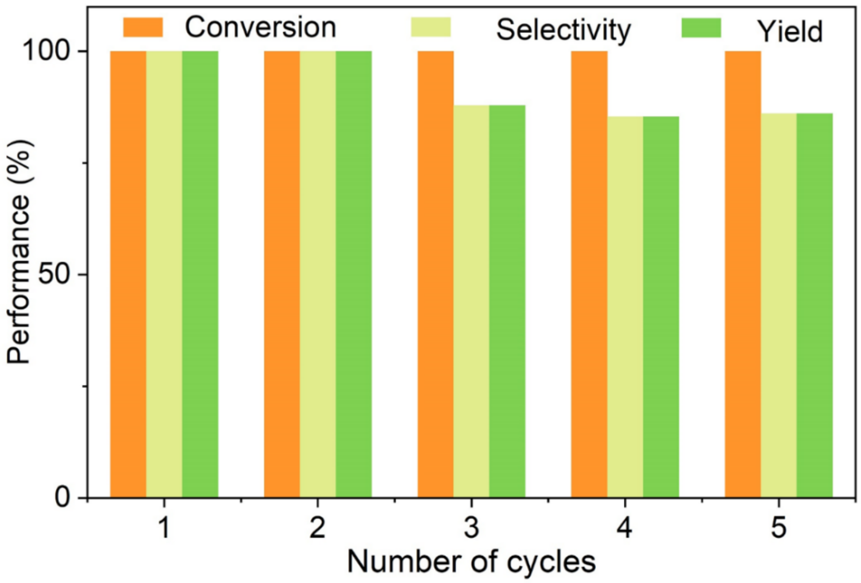

Supplementary Fig. 13: Recycling performance of the catalyst for the photocatalytic reduction nitrobenzene. Reaction conditions: 1 mmol of nitrobenzene, 1 ml of hydrazine hydrate, 2 mg catalyst, light irradiation with continuous stirring at room temperature for 4 h.

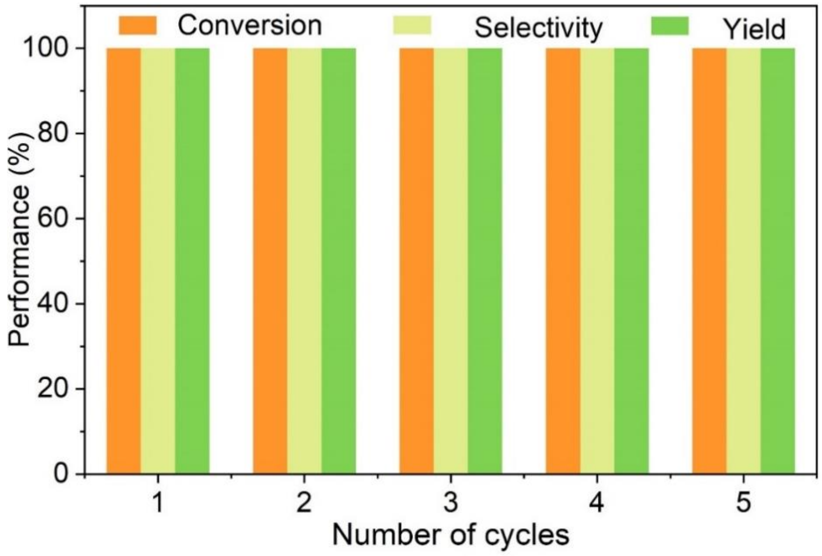

Supplementary Fig. 14: Recycling performance of the catalyst for the photocatalytic reduction nitrobenzene. Reaction conditions: 0.1 mmol of nitrobenzene, 0.05 mL of hydrazine hydrate, 10 mg catalyst, light irradiation with continuous stirring at room temperature for 4 h.

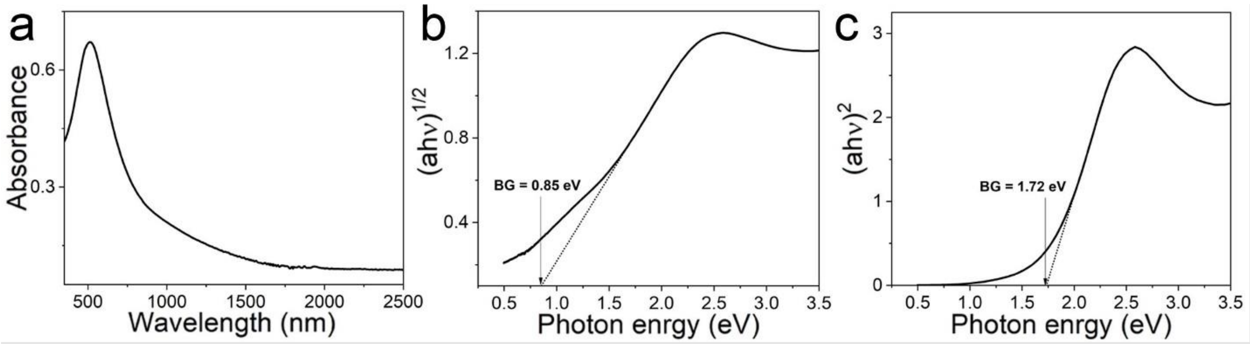

Supplementary Fig. 15: Band-gap determination. **(a)** UV-Vis-NIR absorption spectrum of $CuFeS_2$ NCs film deposited on glass substrate. Tauc plots of $CuFeS_2$ NCs for the determination of the **(b)** direct band gap between the valence band and intermediate band and for the **(c)** indirect band gap between the intermediate band and the conduction band.

EPR Section.

2.1.Reactivity of hydrazine under UV light.

The chemical stability of the hydrazine/water solution under UV light (@325 nm) has been followed by monitoring *in situ*, by light induced EPR technique (LEPR), the electronic changes associated to its spin state, which corresponds in the initial form to the closed-shell S=0 specie. Results from the measurements are shown in Supplementary Fig. 15a-c. As soon as UV light is applied to the sample kept frozen in the cavity resonator (see Supplementary Fig. 16b, in particular), a strong symmetric signal develops around g = 2.000 (Supplementary Fig. 16c), which is associated with the formation of a radical specie. The observed signal lacks any resolved ^{14}N nuclear hyperfine term (I_N) and can be tentatively associated to the formation of a trapped $^{\bullet}OOH$ radical within the frozen water matrix under UV light (NH_2-NH_2 + O_2 (in H_2O) $\rightarrow$ NH=NH + 2 $HOO^{\bullet}$ + $2H^+$). When UV-light was cut off (after 450 s of *in situ* irradiation time), the EPR signal remained well detectable at low temperature (T = 80 K), without showing any alterations in its intensity over 1200 s of signal-detection window. However, it disappeared upon thawing.

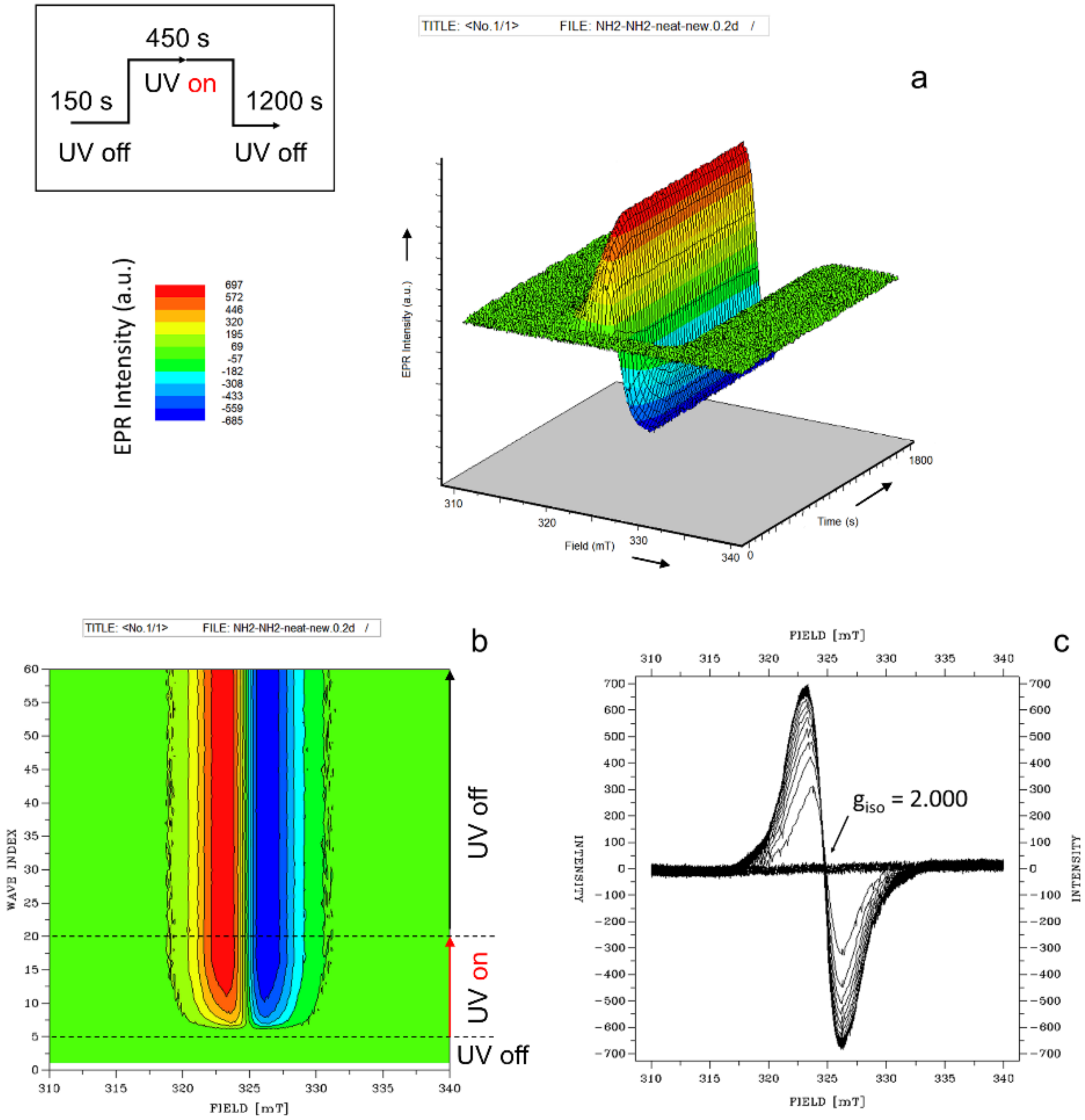

Supplementary Fig. 16: (a-c) X-band (9.084 GHz) LEPR measurements of the NH_2-NH_2 (in water) solution under UV light irradiation. Note that each wave index (spectrum) in panel a and b corresponds to 30 sec of sequential acquisition time. Experimental parameters: 100 kHz modulation frequency, 0.2 mW applied microwave power, 0.03 s time constant, 1.0 mT modulation width, T = 80 K. The LEPR sequence used (time t for the UV-off-on-off process) is shown on the upper left-box.

2.2.Reactivity of nitrobenzene and nitrobenzene/hydrazine mixture under UV light.

While neat nitrobenzene does not show any detectable EPR signal, both in dark and under application of UV light (@325 nm), (Supplementary Fig. 17a, green spectrum), combination of 1 to 1 mixture (vol/vol) of reducing agent (hydrazine) and reactant (nitrobenzene) shows, on the contrary, that even under ambient light a radical specie is detected by EPR in the frozen solution (Supplementary Fig. 17b, blue spectrum). This specie is different from the radical signal detected from the neat hydrazine solution shown in Supplementary Fig. 16 (only seen under UV light). In particular, as highlighted in Supplementary Fig. 16b, the resonance signal evidenced a clear g-anisotropy, which can be linked to the ^{14}N nuclear hyperfine component acting on the electron spin moment. Therefore, an electron transfer occurs between hydrazine and nitrobenzene, leading to formation of a radical intermediate, most probably centered on the nitrobenzene. After *in situ* application of UV light (@325 nm) to the frozen sample kept inside the cavity resonator, a more complex EPR signal appears (Supplementary Fig. 19a, red-spectrum), which can be interpreted as combination of doublet (S=1/2) and triplet species (S=1, D ~17 mT). Overall, it is found that hydrazine molecules do react with the nitrobenzene substrate even in absence of catalysts, but such reaction leads to the formation of various spin active intermediates/species, especially when high energy (hʋ) is provided from the light source, and likely results into an admixture of variously reduced aromatic by-products. Further analysis of the interaction between hydrazine and nitrobenzene without catalyst goes beyond the scope of the current investigation. Importantly these species, as shown from the gas chromatography of a control reaction between nitrobenzene and hydrazine results only into a 5 % conversion of a mixture of products (Supplementary Fig. 11).

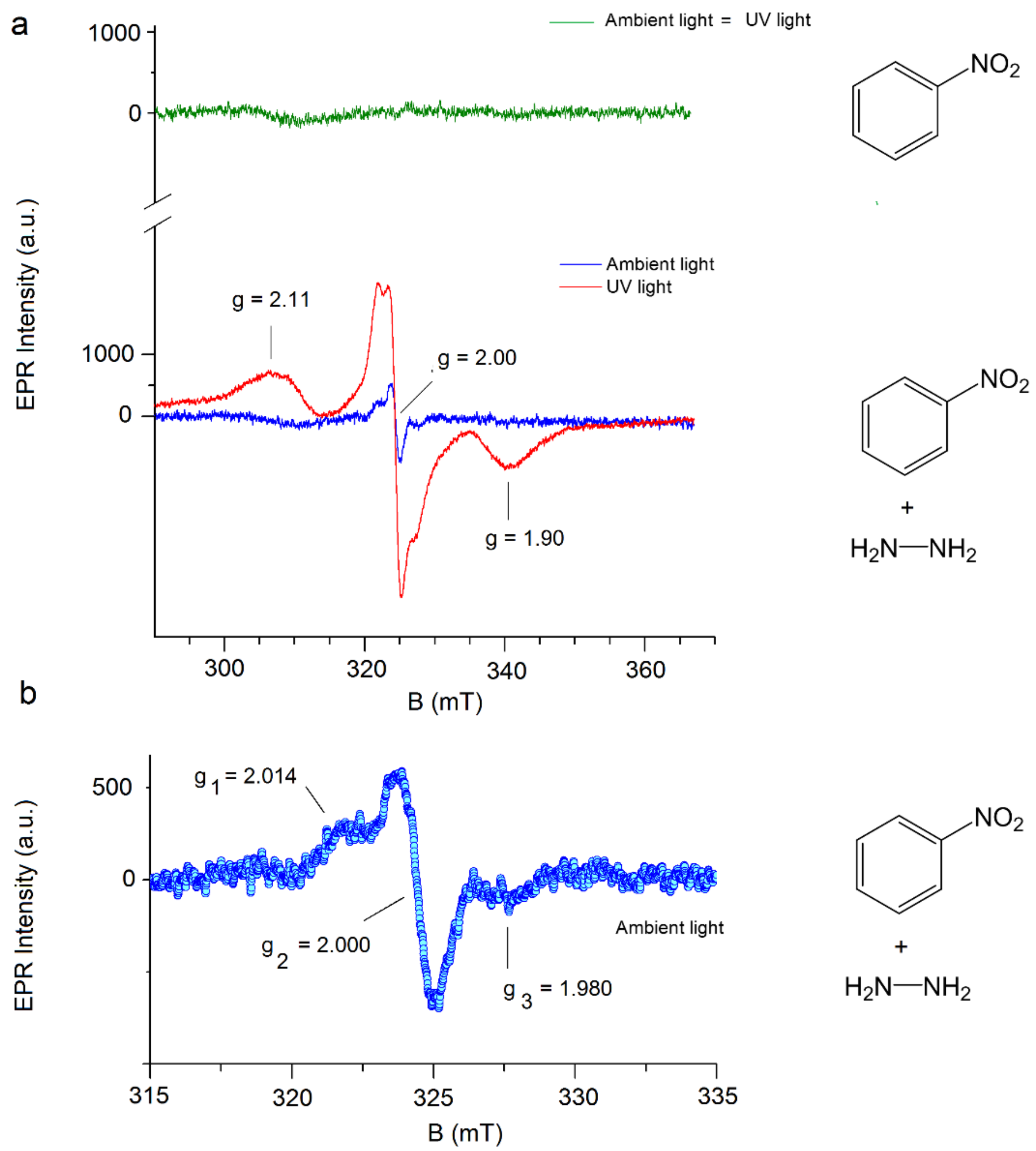

Supplementary Fig. 17: Panel **(a)** X-band (9.076-9.080 GHz) LEPR measurements of the nitrobenzene solution under UV light irradiation (@325 nm, olive line) compared to mixture of hydrazine/nitrobenzene under dark (blue spectrum) and under UV light (red spectrum). Panel **(b)** shows magnification of the blue spectrum shown in Panel (a). Experimental parameters: 100 kHz modulation frequency, 0.4 mW applied microwave power, 0.03 s time constant, 1.0 mT modulation width, T = 80 K, sweep time of 2 min.

2.3. Reactivity of neat nitrobenzene, neat hydrazine and nitrobenzene/hydrazine mixture in presence of the $CuFeS_2$ NCs catalysts.

Further insights on the mechanism of nitroarene reduction into aniline promoted by $CuFeS_2$ NCs with hydrazine were obtained by *in situ* continuous wave (CW) light-induced-EPR technique (LEPR). The changes on the resonance signatures of the catalyst during *in situ* irradiation (@325 nm = 3.8149 eV, 200 mW power) were monitored by probing the frozen-matrix solutions (T = 120 K) of the catalyst dispersed in water, neat nitrobenzene, neat hydrazine and their combinations. Supplementary Fig. 18a shows the EPR traces recorded for the $CuFeS_2$ NCs catalyst suspended in water (~ 2 mg/mL) without (black line) and under (orange line) continuous UV-light irradiation (5 min) of the frozen sample. In water, $CuFeS_2$ NCs exhibits a very broad but nearly symmetric resonance signal (ΔB_{pp} ~ 79 mT) cantered at g_{avg} = 2.04, which arises from the Fe spin containing sites. The formal oxidation states for Fe and Cu cations in chalcopyrite has been predicted to be $Cu^{1+}Fe^{3+}(S^{2-})_2$, thus the Cu sites are EPR silent; however, the observed EPR signal of $CuFeS_2$ NCs is not typically encountered in isolated d^5 Fe(III) systems, in any adopted Fe spin configuration, S=1/2, 3/2 and 5/2, and also it strongly departs from the signatures observed in iron containing nanosized spin systems, such as magnetite and maghemite (Supplementary Fig. 18b, 10-20 nm particles). Furthermore, the resonance signal of $CuFeS_2$ NCs in water does not change, both shape and intensity, under continuous UV-light irradiation, an indication for the red-ox and chemical stability of the catalyst dispersed in water medium.

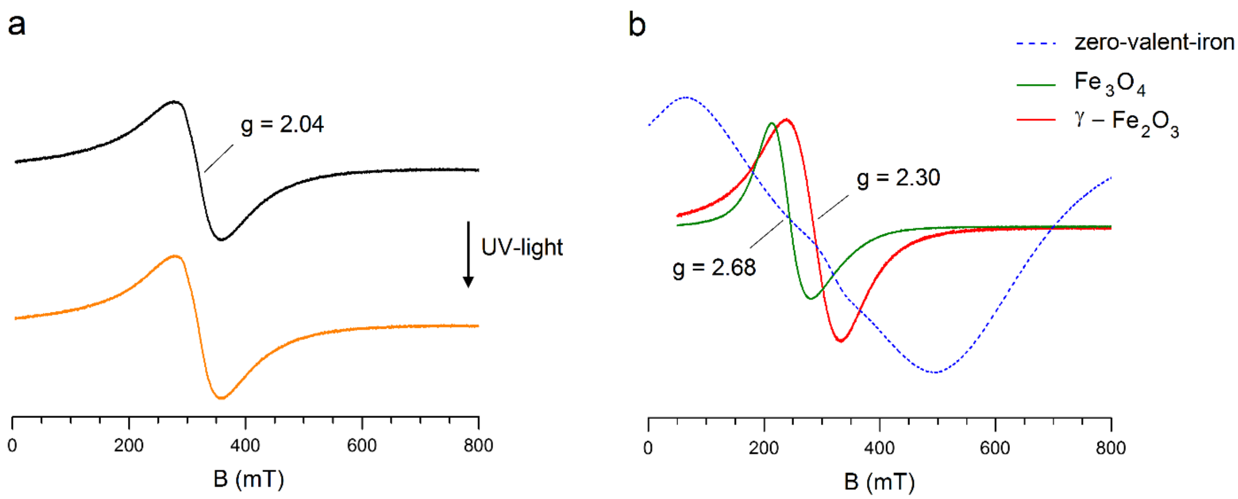

Supplementary Fig. 18: Panel (a). X-band (9.08-9.09 GHz) CW-LEPR signals observed for the $CuFeS_2$ catalyst dispersed in water. The black-spectra is associated to the experiment performed under dark conditions and the orange spectra is that recorded under *in situ* irradiation of the frozen sample with UV-light (@325 nm, 200 mW). Experimental parameters:100 kHz modulation frequency, 1.0 mT modulation width, 2.5 x 100 Gain, 8 min sweep time, 0.03 s time constant, 0.2 mW applied power, T = 120 K. Panel b). X-band EPR spectra of Fe_3O_4 nanoparticles (dispersed in H_2O/glycerol), of maghemite (γ-Fe_2O_3) nanoparticles (dispersed in H_2O/glycerol), and zero-valent-iron (n-ZVI) nanoparticles (dry sample, kept under N_2). The EPR spectra were recorded at T = 120 K. Experimental conditions: Frequency 9.164–9.167 GHz, 100 kHz modulation frequency, 0.8 mT modulation width, 0.02–0.05 mW microwave power, 0.03 s time constant and 8 min sweep time.

The EPR signal recorded in dark conditions of CuFeS$_2$ NCs suspended in neat nitrobenzene (~ 2 mg/mL) is broader (B_{pp} ~ 93 mT) than that observed in water, slightly asymmetric, whilst exhibiting similar g_{avg} value of 2.04 (Supplementary Fig. 18, black line); thus, perturbation of the ligand field associated to the Fe sites occur, suggesting an interaction of the Fe sites with the substrate. *In situ* application of UV-light (orange spectrum, Supplementary Fig. 19) did not show any change in the resonance features compared to the spectrum recorded under dark conditions. Similar results were obtained for $CuFeS_2$ NCs suspended in both water/nitrobenzene mixture (1/1, vol/vol). Therefore, within the reduction process of nitrobenzene into aniline, protons (H^+) as well as electrons (e^-) are not provided directly by activated water molecules that might bind/interact with $CuFeS_2$ NCs.

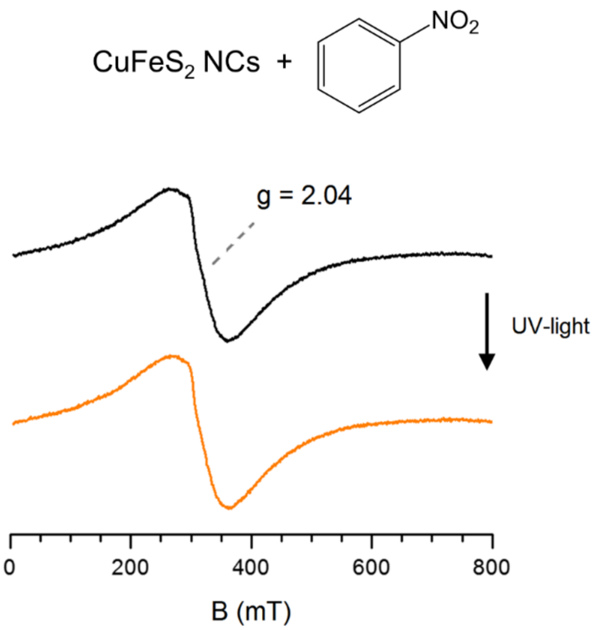

Supplementary Fig. 19: X-band (9.08-9.09 GHz) CW-LEPR signals observed for the $CuFeS_2$ catalyst dispersed in nitrobenzene. The black-spectra is associated to the experiment performed under dark conditions and the orange spectra is that recorded under *in situ* irradiation of the frozen sample with UV-light (@325 nm, 200 mW). Experimental parameters:100 kHz modulation frequency, 1.0 mT modulation width, 2.5 x 100 Gain, 8 min sweep time, 0.03 s time constant, 0.2 mW applied power, $T = 120$ K.

The EPR spectra recorded for the suspension of <u>$CuFeS_2$ NCs in hydrated hydrazine solution</u> (50% water) in dark conditions and under *in situ* application of UV light (@325 nm, 200 mW) are shown in Supplementary Fig. 20 (black line and orange line, respectively). Under dark conditions, the EPR signal observed in the frozen mixture did resemble that witnessed for $CuFeS_2$ NCs suspended in nitrobenzene (Supplementary Fig. 19), and thus the similarly estimated g_{avg} value of 2.04 was extracted. However, it does show significantly less broad resonance line ($\Delta B_{pp} \sim 61$ mT). Immediately following UV light irradiation, a new signal appeared at $g_{avg} \sim 2.000$ (Supplementary Fig. 20, orange line; see also Supplementary Fig. 21a-c). This signal did not clearly change in both shape and relative intensity even after 30 min of irradiation time inside the EPR cavity. While the $g_{avg} \sim 2.000$ signal is reminiscent of the spectrum observed in neat hydrazine under UV light (Supplementary Fig. 16), it does exhibit some differences. Closer inspection of this signal was

obtained by recording its EPR signatures by signal accumulation, in a narrow magnetic field-sweep range, using weaker applied microwave power and smaller modulation-width (Supplementary Fig. 21d). This spin containing specie clearly belongs to a photoexcited spin state generated under UV light formed by hydrazine and $CuFeS_2$ NCs system. However, differing from the case of the detected EPR signal of neat hydrazine under UV, here the EPR signal associated to the photoexcited spin active specie shows several additional resonances, whose contribution may arise from both the effective interaction of the electron spin moment with nuclear hyperfine terms (*e.g.* from ^1H centres). It is excluded that these resolved resonances come from part of Cu(I) oxidized to Cu(II), because the observed splitting (~ 1.0 mT) is too small to arise from the copper nuclear hyperfine term (I= 3/2). We may suggest that the observed resonance is the overlapped combination of S=1/2 signals from trapped $HOO^{\bullet}$ specie, as seen earlier in Supplementary Fig. 16, and an S=1/2 signals generated by various forms of the hydrazine bound/$CuFeS_2$ NCs system, with tentative structures given in the drawings shown in Supplementary Fig. 21e.

Upon dispersion of $CuFeS_2$ NCs in water/nitrobenzene/hydrazine mixture (~ 2 mg, 1 mL, ratios 1/1/1, vol/vol/vol), it was noticed that even under normal ambient light, evolution of N_2 from the vial containing the catalyst and the water solvent plus reactants (nitrobenzene and hydrazine) occurred very fast; the same effect has been observed earlier to occur in the hydrazine/nitrobenzene mixture (see Supplementary Fig. 17). Therefore, $CuFeS_2$ NCs were pre-suspended in water/nitrobenzene (~2 mg/mL), transferred to an EPR tube and an aliquot of hydrazine was then added inside the EPR tube, followed by fast freeze quenching into liquid nitrogen bath. The fast-quenched EPR spectrum of $CuFeS_2$ NCs in water/nitrobenzene/hydrazine recorded without *in situ* application of UV-light is shown in Supplementary Fig. 22a (black line), and magnification of the magnetic field region around g = 2.000 is given in Supplementary Fig. 22b. A very strong signal

appears in this region, which increases in intensity as soon as UV light is applied to the frozen sample inside the cavity resonator (Supplementary Fig. 22a-b, orange line). See also the EPR spectra shown in Supplementary Fig. 23a-c, obtained by fast LEPR acquisition procedure at T= 77K. This type of signal is highly reminiscent of those expressed by nitroxide-based radicals (-N-O$^{\bullet}$) and can be associated to the three-electron reduced intermediate form of the nitrobenzene substrate forming *N*-phenylhydroxylamine radical, as shown in the Supplementary Fig. 23d (inset). The spin-Hamiltonian simulation (2nd order perturbation theory) of this signal is given by the blue-spectrum in Supplementary Fig. 23d, with g_{xx} = 2.0066, g_{yy} = 2.0049, g_{zz}= 2.0020, A_{xx} = 0.44 mT, A_{yy} = 0.44 mT, A_{zz} = 2.80 mT. The presence of such radical species has been further validated by spin-trap technique using POBN as trapping agent (Supplementary Fig. 24 and Supplementary Fig. 25). Therefore, within the catalytic cycle (Supplementary Fig. 26 and Supplementary Fig. 27), which overall involves transfer of 6 electrons (6e^{-}) and 6 protons (6H^{+}) from the reducing agent (hydrazine), the preferred reaction pathway appears to proceed through pathway A, rather than *via* pathway B (Supplementary Fig. 27).

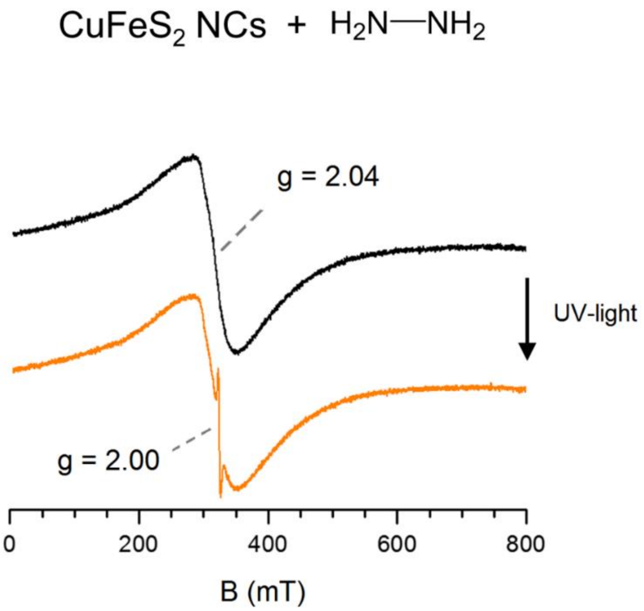

Supplementary Fig. 20: X-band (9.08-9.09 GHz) CW-LEPR signals observed for the $CuFeS_2$ catalyst dispersed in hydrazine. The black-spectra is associated to the experiment performed under dark conditions and the orange spectra is that recorded under *in situ* irradiation of the frozen sample with UV-light (@325 nm, 200 mW). Experimental parameters:100 kHz modulation frequency, 1.0 mT modulation width, 2.5 x 100 Gain, 8 min sweep time, 0.03 s time constant, 0.2 mW applied power, T = 120 K.

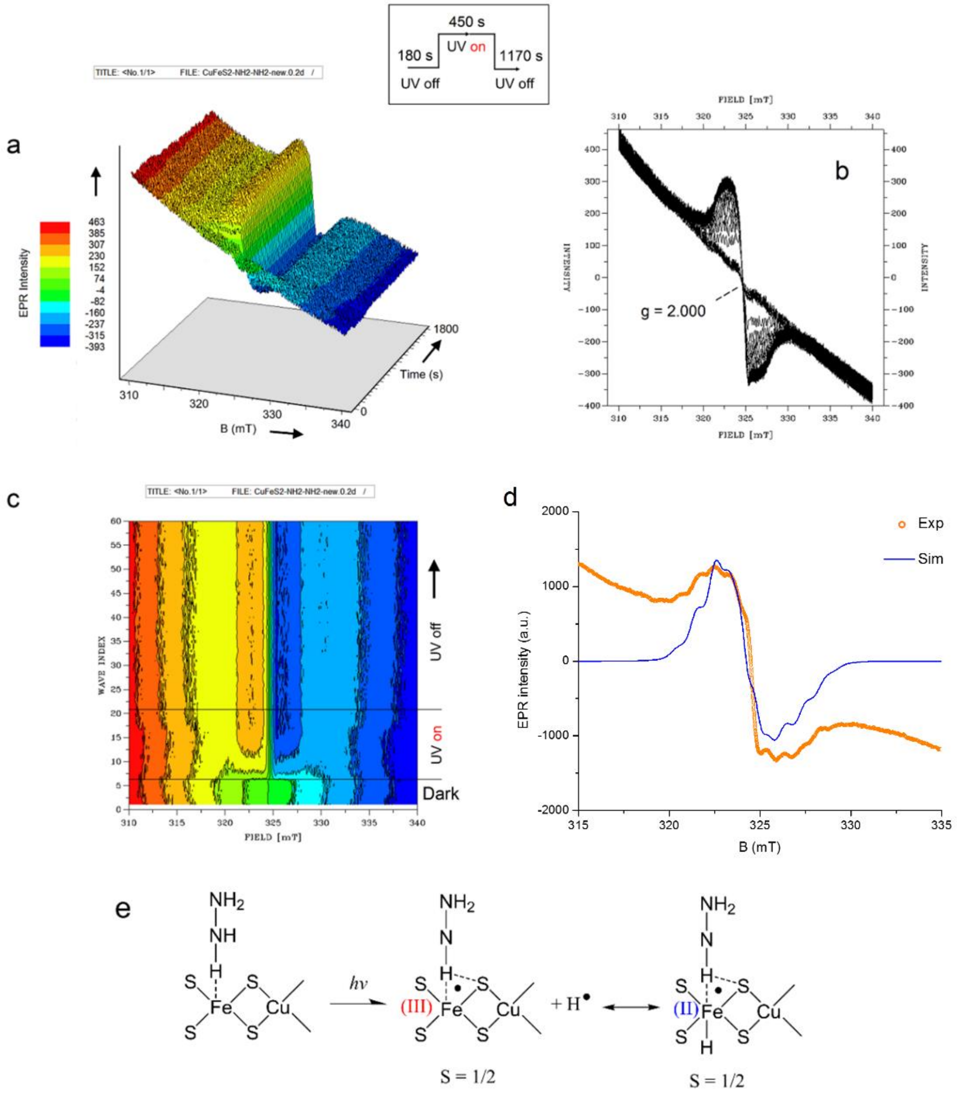

Supplementary Fig. 21: Panel a-c, X-band (9.083 GHz) CW-LEPR signals observed for the $CuFeS_2$ catalyst dispersed in hydrazine. The UV light off→ light on→ light off time sequence is shown in the upper box (180 s, light-off; 450 s, light-on, 1170 s, light-off). Experimental parameters:100 kHz modulation frequency, 1.0 mT modulation width, 1.0 x 100 Gain, 30 s sweep time each spectrum, 0.03 s time constant, 0.2 mW applied power, T = 80 K. Panel d) shows the EPR spectrum recorded under UV light, of the spin active intermediate with the following acquisition parameters: 9. 077 GHz frequency, 100 kHz modulation frequency, 0.5 mT modulation width, 4.0 x 100 Gain, 2 min sweep time, 0.03 s time constant, 0.1 mW applied power, 7 scans accumulated, T = 80 K. Panel e) shows the possible structures associated to the S=1/2 spin species formed with EPR signal given in panels (a-d).

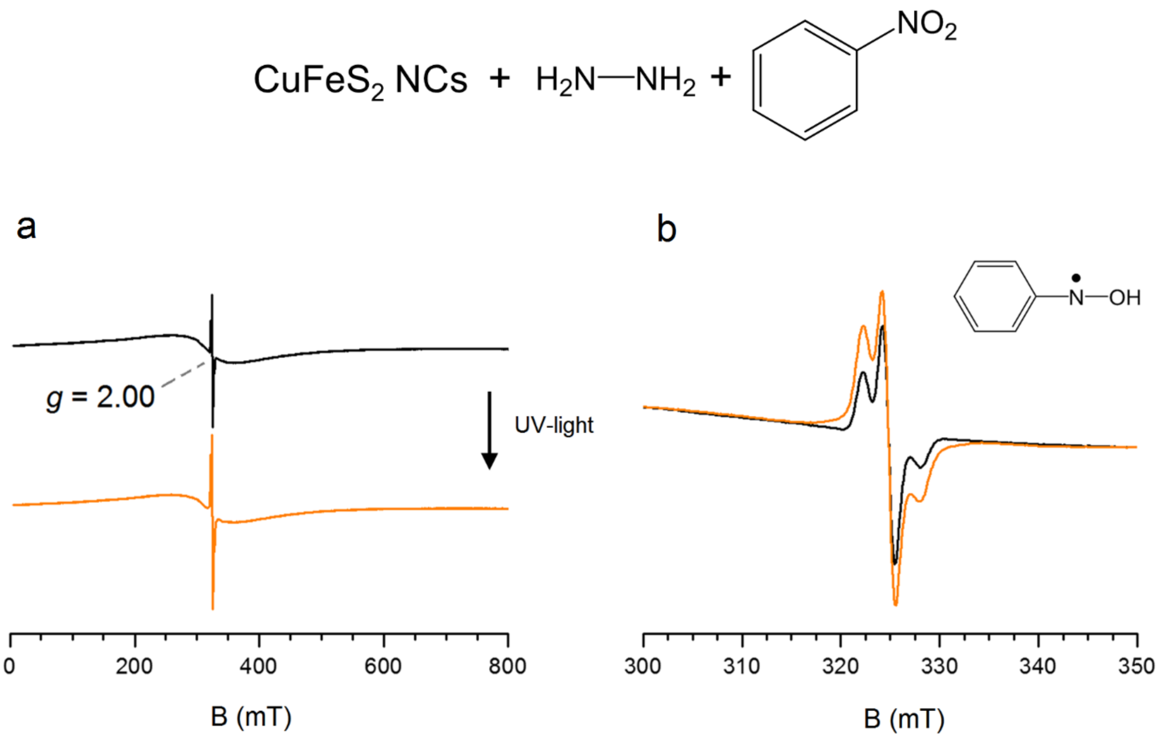

Supplementary Fig. 22: Panel a: X-band (9.08-9.09 GHz) CW-LEPR signals observed for the $CuFeS_2$ catalyst dispersed in hydrazine together with the nitrobenzene substrate. The black-spectra is associated to the experiment performed under ambient light conditions and the orange spectra is recorded under *in situ* irradiation of the frozen sample with UV-light (@325 nm, 200 mW). Experimental parameters:100 kHz modulation frequency, 1.0 mT modulation width, 2.5 x 100 Gain, 8 min sweep time, 0.03 s time constant, 0.2 mW applied power, T = 120 K. Panel b) shows signal magnification around the g = 2.000 region of the EPR resonance spectra given in panel a). The molecular structure shown in panel b) corresponds to the detected *N*-phenylhydroxylamine radical intermediate.

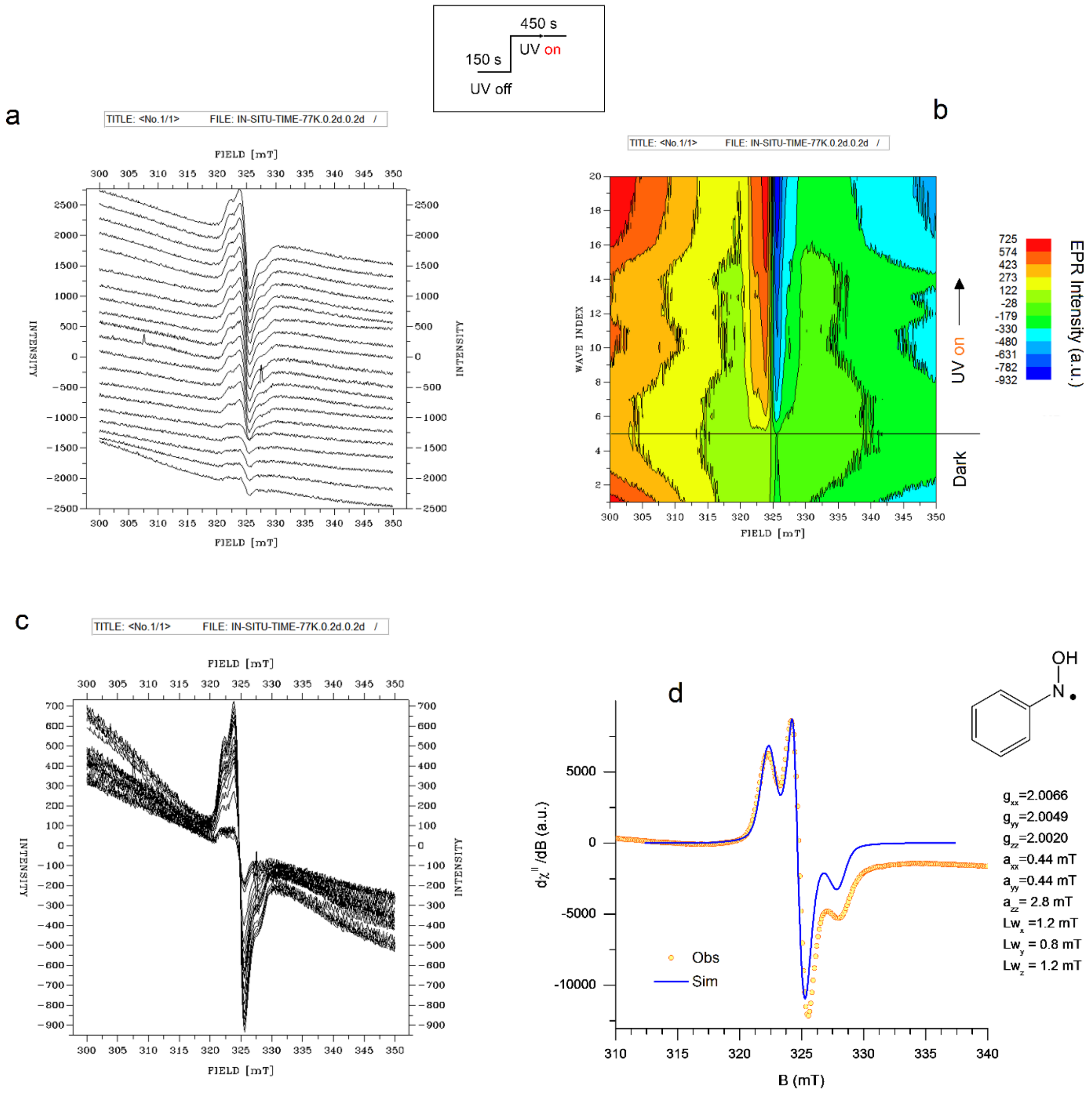

Supplementary Fig. 23: (**a-c**) Time evolution of the X-band (9.086 GHz) CW-LEPR signals observed for the $CuFeS_2$ catalyst dispersed in hydrazine/water solution and nitrobenzene. The UV light off→ light on time sequence is shown in the upper box (150 s, light-off; 450 s, light-on). Experimental parameters:100 kHz modulation frequency, 1.0 mT modulation width, 1.0 x 100 Gain, 30 s sweep time each spectrum, 0.03 s time constant, 0.2 mW applied power, T = 77 K. Panel (**d**) shows the EPR spectrum recorded under UV light depicted earlier in Supplementary Fig. 21 (orange line) together with its spin-Hamiltonian simulation (blue line) correspondent to the *N*-phenylhydroxylamine radical intermediate.

2.4.Spin trap experiments as monitored by the CW-EPR technique.

The spin-trap molecule, α-4-pyridyl-1-oxide-N-tert-butylnitrone (POBN) was used to validate the presence of radical species arising from the catalytic conversion of nitrobenzene to aniline under UV-light irradiation. Experimentally, solid POBN was added to a water suspension containing the $CuFeS_2$ NCs catalyst, hydrazine, and nitrobenzene (concentration of POBN = 9 mg/ mL) (Supplementary Fig. 24a), and the solution was stirred at room temperature for 5 min under constant UV light irradiation (at 325 nm). Then, 0.2 mL of this mixture were transferred into an EPR tube and fast frozen at 77 K in liquid nitrogen prior to the EPR measurement. A blank experiment, under the same experimental conditions reported above, using only hydrazine in water, nitrobenzene and POBN without the presence of a catalyst was also performed as control sample; the EPR results are shown in the Supplementary Fig. 24b. The molecular structure depicted in blue color (Supplementary Fig. 24a) indicates the trapped *N*-phenylhydroxylamine radical species using the POBN probe.

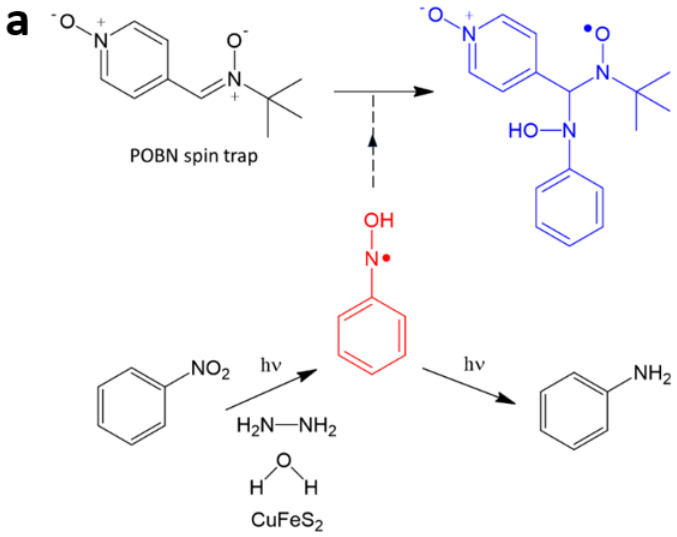

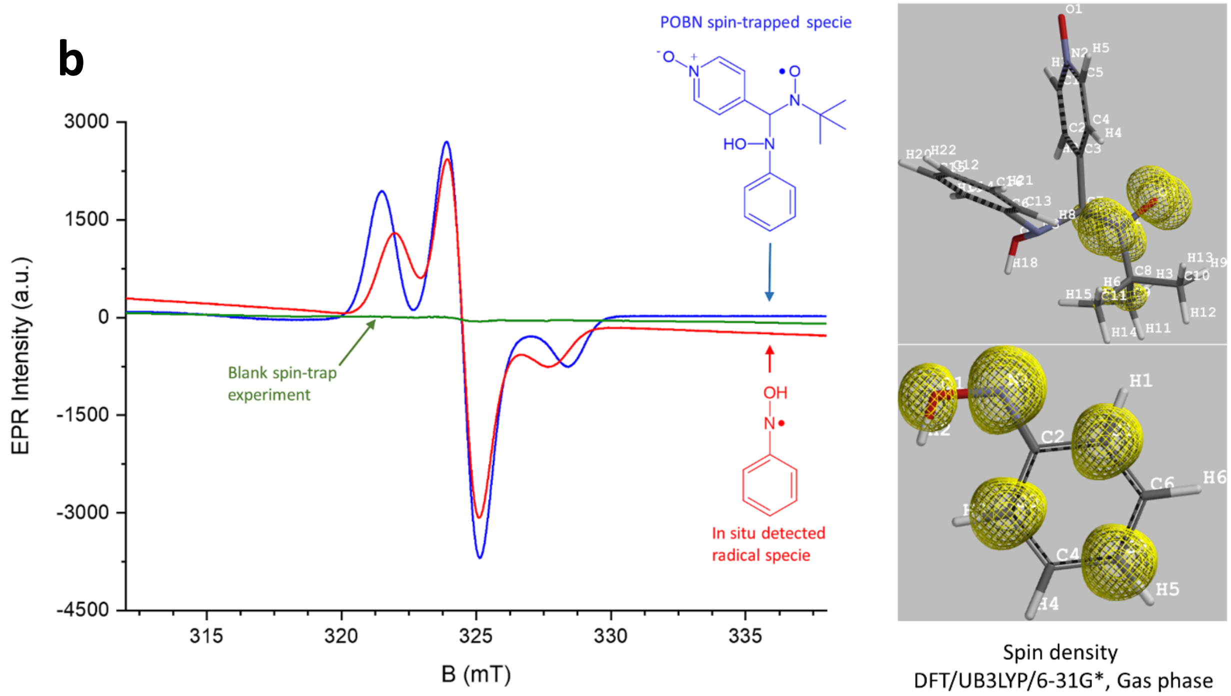

Supplementary Fig. 24: (a) Description of the spin trap experiment. **(b)** X-band (9.08-9.09 GHz) CW-EPR signals of the trapped spin specie (blue EPR trace) observed at 77 K (0.2 mW applied power) as detected from the admixture POBN/NH_2-NH_2/nitrobenzene in presence of $CuFeS_2$ catalyst exposed for 5 min under UV-light. The structure of the POBN spin trapped radical adduct is shown in blue color by the chemical drawing. The EPR plot shows, for comparison, the resonance features (recorded at 77 K) of the radical species (*N*-phenylhydroxylamine radical) detected during *in situ* UV-light irradiation of the NH_2-NH_2/nitrobenzene solution in the presence of $CuFeS_2$ (molecule shown in red, also in red color is shown its EPR signal). The EPR signal in green color shows the result obtained from the blank experiment, performed under identical conditions, using the admixture of POBN/hydrazine/nitrobenzene without the catalyst. The g-tensor parameters of the trapped POBN radical adduct are similar to those derived from the *in situ* detected *N*-phenylhydroxylamine radical but expresses a substantial alteration in the anisotropic N hyperfine splitting terms (hfc, A_{zz} of 3.4 mT, A_{xx} and A_{yy} of 0.46 mT). This experimental result is also validated by theoretical calculations (DFT/UB3LYP/6-31G*) *via* the analysis of the spin density distribution (theoretical models drawn on the right).

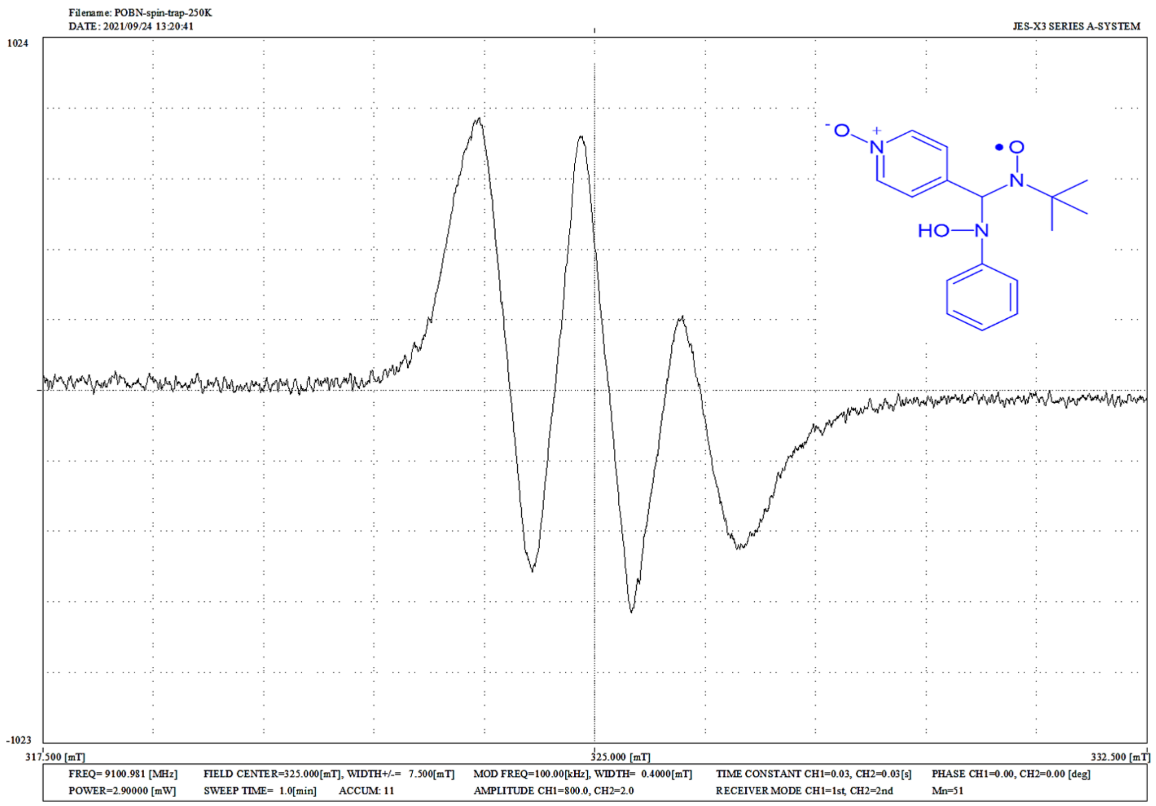

Supplementary Fig. 25: X-band (9.10 GHz) CW-EPR signals of the trapped spin specie observed at 250 K (2.9 mW applied power) as detected from the admixture POBN/NH_2-NH_2/nitrobenzene in presence of $CuFeS_2$ catalyst exposed for 5 min under UV-light. The isotropic hyperfine coupling constant A_N = 1.4 mT.

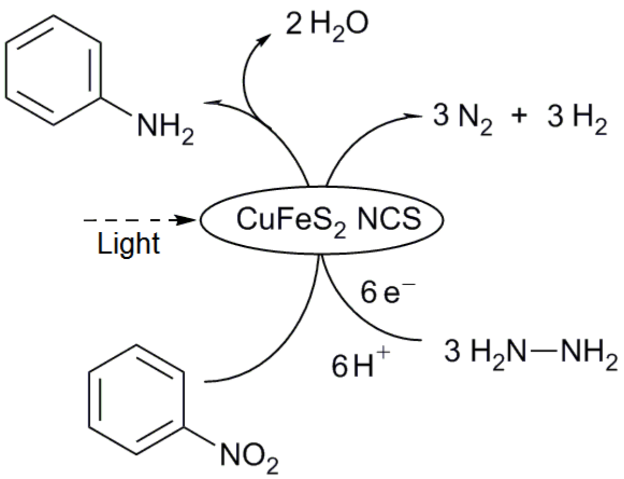

Supplementary Fig. 26: General reaction mechanism for the nitrobenzene reduction using hydrazine and catalyst under visible light, generally observed for this this reaction.

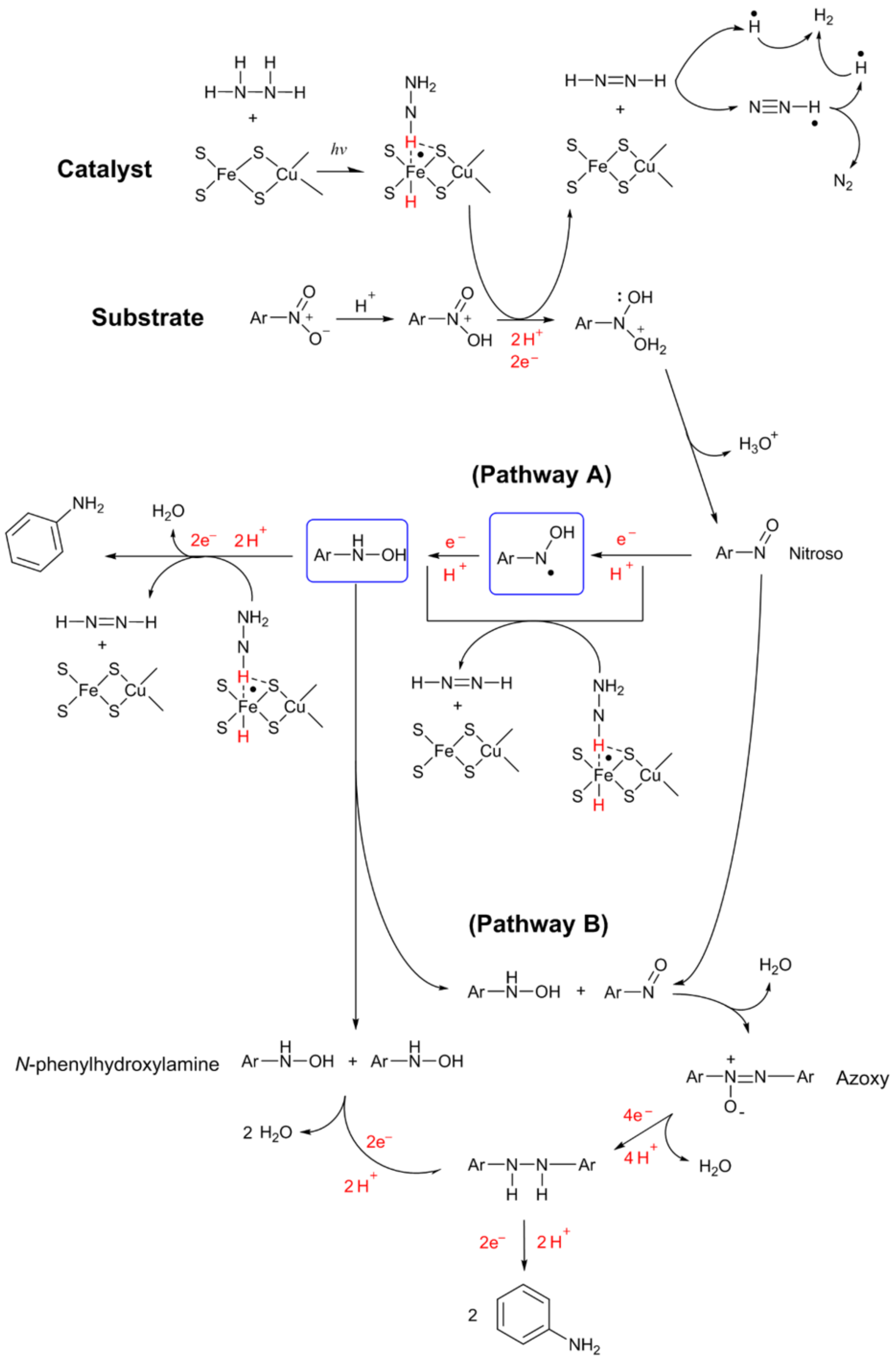

Supplementary Fig: 27: Details of the reaction mechanism for nitrobenzenereduction using the $CuFeS_2$ catalyst in presence of hydrazine hydrate under light with key intermediates experimentally observed enclosed by blue boxes.

2.5. Identification of intermediates by GC before complete conversion of nitrobenzene

Gas chromatographs (GC) during the progress of the photocatalytic reaction, revealing the presence of phenylhydroxylamine as the only intermediate (Supplementary Fig. 28). Phenylhydroxylamine's identification, being a major intermediate in the nitrobenzene reduction pathway, also suggests Path (A) as the dominant one, in keeping to the previously discussed detection of the nitroxyl radical intermediate. Similar GC performed under thermal conditions in the dark revealed the complete absence of phenylhydroxylamine and the significant presence of azoxybenzene (the precursor of hydrazobenzene, Supplementary Figure 27), signaling the dominance in this case of Path (B), since azoxybenzene and hydrazobenzene are connected to Path (B). This finding, verifies the prevailing pathway (A) in presence of light, but also unveils the key role of the photocatalytic process, by boosting the reaction *via* activation of the proton and electron donor (hydrazine), but also defining the reaction coordinate by favoring the most direct formation of hydroxylamine *via* Pathway (A).

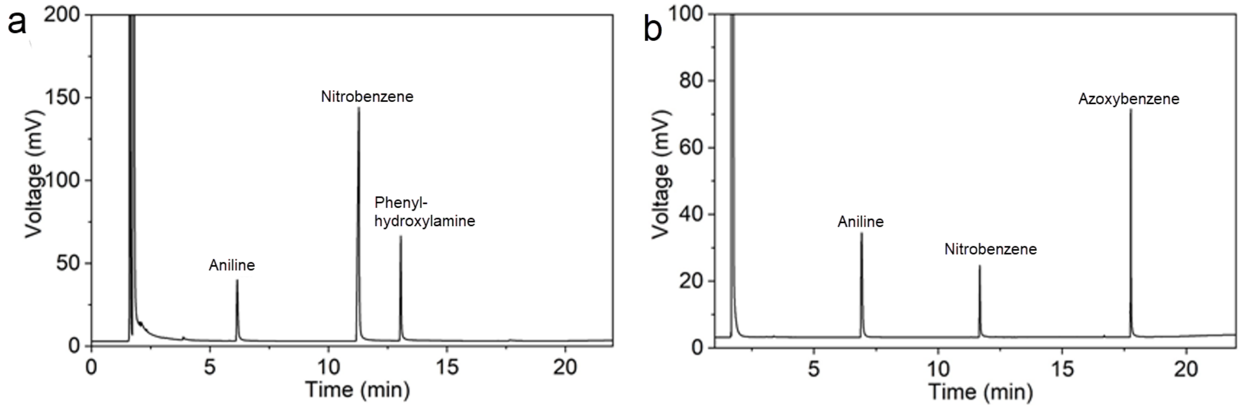

Supplementary Fig. 28: GC graphs during nitrobenzene reduction and before completion **(a)** in the presence of light and **(b)** without light under forced heating at 60 °C with continuous stirring at room temperature for 1 h.

Supplementary References

1 Nag, A. *et al.* Metal-free Inorganic Ligands for Colloidal Nanocrystals: S^{2-}, HS^-, Se^{2-}, HSe^-, Te^{2-}, HTe^-, TeS_3^{2-}, OH^-, and NH_2^- as Surface Ligands. *J. Am. Chem. Soc.* **133**, 10612-10620 (2011).

2 Yang, Y. W. *et al.* High-quality Cu_2ZnSnS_4 and $Cu_2ZnSnSe_4$ nanocrystals hybrid with ZnO and $NaYF_4$: Yb, Tm as efficient photocatalytic sensitizers. *Appl. Catal. B* **200**, 402-411 (2017).

3 Konidakis, I. *et al.* Improved Charge Carrier Dynamics of $CH_3NH_3PbI_3$ Perovskite Films Synthesized by Means of Laser-Assisted Crystallization. *ACS Appl. Energy. Mater.* **1**, 5101-5111 (2018).

4 Konidakis, I. *et al.* Erasable and rewritable laser-induced gratings on silver phosphate glass. *Appl. Phys. A Mater. Sci. Process.* **124** (2018).

5 Serpetzoglou, E. *et al.* In situ monitoring of the charge carrier dynamics of $CH_3NH_3PbI_3$ perovskite crystallization process. *J Mater. Chem. C Mater.* **7**, 12170-12179 (2019).

6 Fukui, M., Koshida, W., Tanaka, A., Hashimoto, K. & Kominami, H. Photocatalytic hydrogenation of nitrobenzenes to anilines over noble metal-free TiO_2 utilizing methylamine as a hydrogen donor. *Appl. Catal. B* **268** (2020).

7 Zhang, H. *et al.* P/N co-doped carbon derived from cellulose: A metal-free photothermal catalyst for transfer hydrogenation of nitroarenes. *Appl. Surf. Sci.* **487**, 616-624 (2019).

8 Zhang, S. J. *et al.* Photocatalytic organic transformations: Simultaneous oxidation of aromatic alcohols and reduction of nitroarenes on $CdLa_2S_4$ in one reaction system. *Appl. Catal. B* **233**, 1-10 (2018).

9 Xiao, Q. *et al.* Alloying Gold with Copper Makes for a Highly Selective Visible-Light Photocatalyst for the Reduction of Nitroaromatics to Anilines. *ACS Catal.* **6**, 1744-1753 (2016).

10 Zhang, S. J. *et al.* Ultra-low content of Pt modified CdS nanorods: Preparation, characterization, and application for photocatalytic selective oxidation of aromatic alcohols and reduction of nitroarenes in one reaction system. *J. Hazard. Mater.* **360**, 182-192 (2018).

11 Karthik, R. *et al.* A Study of Electrocatalytic and Photocatalytic Activity of Cerium Molybdate Nanocubes Decorated Graphene Oxide for the Sensing and Degradation of Antibiotic Drug Chloramphenicol. *ACS Appl. Mater. Interfaces* **9**, 6547-6559 (2017).

12 Zhang, L. Q. *et al.* Highly active TiO_2/g-C_3N_4/G photocatalyst with extended spectral response towards selective reduction of nitrobenzene. *Appl. Catal. B* **203**, 1-8 (2017).

13 Xu, S. D., Tang, J. H., Zhou, Q. W., Du, J. & Li, H. X. Interfacing Anatase with Carbon Layers for Photocatalytic Nitroarene Hydrogenation. *ACS Sustain. Chem. Eng.* **7**, 16190-16199 (2019).

14 Challagulla, S., Tarafder, K., Ganesan, R. & Roy, S. Structure sensitive photocatalytic reduction of nitroarenes over TiO_2. *Sci. Rep.* **7** (2017).

15 Kumar, A., Kumar, P., Paul, S. & Jain, S. L. Visible light assisted reduction of nitrobenzenes using $Fe(bpy)_3^{+2}$/rGO nanocomposite as photocatalyst. *Appl. Surf. Sci.* **386**, 103-114 (2016).

16 Kumar, A., Paul, B., Boukherroub, R. & Jain, S. L. Highly efficient conversion of the nitroarenes to amines at the interface of a ternary hybrid containing silver nanoparticles doped reduced graphene oxide/graphitic carbon nitride under visible light. *J. Hazard. Mater.* **387** (2020).

17 Gao, W. Z., Xu, Y., Chena, Y. & Fu, W. F. Highly efficient and selective photocatalytic reduction of nitroarenes using the Ni_2P/CdS catalyst under visible-light irradiation. *Chem. Commun.* **51**, 13217-13220 (2015).

18 Kong, L., Mayorga-Martinez, C. C., Guan, J. G. & Pumera, M. Fuel-Free Light-Powered TiO_2/Pt Janus Micromotors for Enhanced Nitroaromatic Explosives Degradation. *ACS Appl. Mater. Interfaces* **10**, 22427-22434 (2018).

19 Song, Y. J. *et al.* Selective Photocatalytic Synthesis of Haloanilines from Halonitrobenzenes over Multifunctional AuPt/Monolayer Titanate Nanosheet. *ACS Catal.* **8**, 9656-9664 (2018).

20 Kaur, M. & Nagaraja, C. M. Template-Free Synthesis of $Zn_{1-x}Cd_xS$ Nanocrystals with Tunable Band Structure for Efficient Water Splitting and Reduction of Nitroaromatics in Water. *ACS Sustain. Chem. Eng.* **5**, 4293-4303 (2017).
21 Cui, J. B. *et al.* Near-Infrared Plasmonic-Enhanced Solar Energy Harvest for Highly Efficient Photocatalytic Reactions. *Nano Lett.* **15**, 6295-6301 (2015).
22 Yu, Z. J. *et al.* Photocatalytic hydrogenation of nitroarenes using $Cu_{1.94}S$-$Zn_{0.23}Cd_{0.77}S$ heteronanorods. *Nano Res.* **11**, 3730-3738 (2018).
23 Chen, P. Q. *et al.* A visible-light-responsive metal-organic framework for highly efficient and selective photocatalytic oxidation of amines and reduction of nitroaromatics. *J. Mater. Chem. A Mater.* **7**, 27074-27080 (2019).
24 Hao, C. H. *et al.* Synergistic Effect of Segregated Pd and Au Nanoparticles on Semiconducting SiC for Efficient Photocatalytic Hydrogenation of Nitroarenes. *ACS Appl. Mater. Interfaces* **10**, 23029-23036 (2018).
25 Jagadeesh, R. V. *et al.* Nanoscale Fe_2O_3-Based Catalysts for Selective Hydrogenation of Nitroarenes to Anilines. *Science* **342**, 1073-1076 (2013).
26 Westerhaus, F. A. *et al.* Heterogenized cobalt oxide catalysts for nitroarene reduction by pyrolysis of molecularly defined complexes. *Nat. Chem.* **5**, 537-543 (2013).
27 Zhou, P. *et al.* High performance of a cobalt-nitrogen complex for the reduction and reductive coupling of nitro compounds into amines and their derivatives. *Sci. Adv.* **3** (2017).
28 Goswami, A. *et al.* Fe(0)-embedded thermally reduced graphene oxide as efficient nanocatalyst for reduction of nitro compounds to amines. *Chem. Eng. J.* **382** (2020).
29 Gong, W. B. *et al.* Nitrogen-Doped Carbon Nanotube Confined Co-N_x Sites for Selective Hydrogenation of Biomass-Derived Compounds. *Adv. Mater.* **31** (2019).
30 Li, W. *et al.* General and Chemoselective Copper Oxide Catalysts for Hydrogenation Reactions. *ACS Catal.* **9**, 4302-4307 (2019).
31 She, W. *et al.* High Catalytic Performance of a CeO_2-Supported Ni Catalyst for Hydrogenation of Nitroarenes, Fabricated via Coordination-Assisted Strategy. *ACS Appl. Mater. Interfaces* **10**, 17487-17487 (2018).
32 Lin, L. L. *et al.* A highly CO-tolerant atomically dispersed Pt catalyst for chemoselective hydrogenation. *Nat. Nanotechnol.* **14**, 354-361 (2019).
33 Wang, C. R., Xue, S. L., Hu, J. Q. & Tang, K. B. Raman, Far Infrared, and Mossbauer Spectroscopy of $CuFeS_2$ Nanocrystallites. *Jpn. J. Appl. Phys.* **48** (2009).
34 Kumar, P., Uma, S. & Nagarajan, R. Precursor driven one pot synthesis of wurtzite and chalcopyrite $CuFeS_2$. *Chem. Commun.* **49**, 7316-7318 (2013).
35 Li, Y. X. *et al.* High-index faceted $CuFeS_2$ nanosheets with enhanced behavior for boosting hydrogen evolution reaction. *Nanoscale* **9**, 9230-9237 (2017).
